# Water-Soluble Salts Based on Benzofuroxan Derivatives—Synthesis and Biological Activity

**DOI:** 10.3390/ijms232314902

**Published:** 2022-11-28

**Authors:** Elena Chugunova, Victoria Matveeva, Alena Tulesinova, Emil Iskanderov, Nurgali Akylbekov, Alexey Dobrynin, Ayrat Khamatgalimov, Nurbol Appazov, Lyazat Boltayeva, Bakhytzhan Duisembekov, Mukhtar Zhanakov, Yulia Aleksandrova, Tatyana Sashenkova, Elena Klimanova, Ugulzhan Allayarova, Anastasia Balakina, Denis Mishchenko, Alexander Burilov, Margarita Neganova

**Affiliations:** 1 Arbuzov Institute of Organic and Physical Chemistry, FRC Kazan Scientific Center, Russian Academy of Sciences, Kazan 420088, Russia; 2 The Kazan National Research Technological University, Kazan 420015, Russia; 3 Kazan Federal University, Kazan 420008, Russia; 4 Laboratory of Engineering Profile “Physical and Chemical Methods of Analysis”, Korkyt Ata Kyzylorda University, Kyzylorda 120014, Kazakhstan; 5 I. Zhakhaev Kazakh Scientific Research Institute of Rice Growing, Kyzylorda 120008, Kazakhstan; 6 Kazakh Scientific Research Institute of Plant Protection and Quarantine Named after Zhazken Zhiembayev LLP, Almaty A30M0H6, Kazakhstan; 7 L.N. Gumilyov Eurasian National University, Astana 010008, Kazakhstan; 8 Institute of Physiologically Active Compounds at Federal Research Center of Problems of Chemical Physics and Medicinal Chemistry, Russian Academy of Sciences, Chernogolovka 142432, Russia; 9 Federal Research Center of Problems of Chemical Physics and Medicinal Chemistry—RAS, Chernogolovka 142432, Russia; 10 Faculty of Fundamental Physical-Chemical Engineering, M.V. Lomonosov—MSU, Moscow 119991, Russia; 11 Biomedical Institute of the Scientific and Educational Center, Moscow Regional State University in Chernogolovka, Mytishchi 141014, Russia

**Keywords:** benzofuroxan, water-soluble salt, cancer, glycolysis, phytopathological analyses of agricultural crops, growth stimulators and regulators, acute toxicity

## Abstract

A series of novel water-soluble salts of benzofuroxans was achieved via aromatic nucleophilic substitution reaction of 4,6-dichloro-5-nitrobenzofuroxan with various amines. The salts obtained showed good effectiveness of the pre-sowing treatment of seeds of agricultural crops at concentrations of 20–40 mmol. In some cases, the seed treatment with salts leads not only to improved seed germination, but also to the suppression of microflora growth. Additionally, their anti-cancer activityin vitrohas been researched. The compounds with morpholine fragments or a fragment of *N*-dimethylpropylamine, demonstrated the highest cytotoxic activity, which is in good correlation with the ability to inhibit the glycolysis process in tumor cells. Two compounds **4e** and **4g** were selected for further experiments using laboratory animals. It was found that the lethal dose of 50% (LD_50_) is 22.0 ± 1.33 mg/kg for **4e** and 13.75 ± 1.73 mg/kg for **4g**, i.e., compound **4e** is two times less toxic than **4g,** according to the mouse model in vivo. It was shown that the studied compounds exhibit antileukemia activity after a single intraperitoneal injection at doses from 1.25 to 5 mg/kg, as a result of which the average lifespan of animals with a P388 murine leukemia tumor increases from 20 to 28%. Thus, the water-soluble salts of benzofuroxans can be considered as promisingcandidates for further development, both as anti-cancer agents and as stimulants for seed germination and regulators of microflora crop growth.

## 1. Introduction

Nitrogen- and oxygen-containing heterocycles have attracted close attention in scientific literature due to their diverse chemistry and wide application as anti-oxidant, anti-cancer [1], antimicrobial agents [2], DNA photocleavage active agents [3], and ligands in complexes with metals [4], etc.

Over the past decades, a huge number of benzofuroxan derivatives have been studied and described, which show a wide range of biological activity [5,6,7,8]. The synthetic potential of benzofuroxans is determined by the easiness of introducing into their structure, while maintaining the furoxan ring, various pharmacophore groups responsible for the appearance of biological activity, as well as obtaining on their basis a wide range of different classes of NO-containing heterocyclic compounds [9,10]. Some benzofuroxans are patented as fungicides against phytopathogenic fungi [11,12]. There are reports of the benzofuroxan compounds’ use as vasodilators [13]. Some of the nitrobenzofuroxans exhibit antitumor [14,15], anti-tuberculosis [16,17], anti-inflammatory [18,19], and antithrombotic properties [20], or are claimed to be effective agents against human and animal pathogens [21,22,23]. Benzofuroxans present a class of heterocyclic compounds that are thiol dependent NO donor agents [24,25,26,27]. Thus, benzofuroxans are considered to be NO-releasing prodrugs whose biological activity is caused by action on sGC, which in turn initiates the formation of the cGMP messenger molecule. Compared to other NO donors, benzofuroxan has certain advantages due to the slow release of the NO molecule, resulting in a longer duration of action.

A series of new amine-containing benzofuroxan derivatives, previously obtained by our research group as a result of the substitution of the chlorine atom in the 4,6-dichloro-5-nitrobenzofuroxan molecule for fragments of various aliphatic and aromatic amines, demonstrated high activity against cancer cell lines and low cytotoxicity against normal cells [28]. Selective cytotoxicity was showed against the cervical carcinoma cell line (M-HeLa) and human breast adenocarcinoma cells (MCF-7), comparable to the reference drug Doxorubicin and significantly superior to Tamoxifen in terms of anticancer effect, as well as high activity against the glioblastoma cell line (T98G).

However, despite the wide range of biological activity of benzofuroxans, the possibility of their use in medicine is limited by their low solubility in water, thereby narrowing the range of their possible dosage forms. All compounds obtained earlier by us were absolutely insoluble in water, which made it difficult, despite their high biological activity, to go from tests on cells in vitro to tests on mice in vivo in the case of studying anticancer activity, or in the case of high fungicidal activity, moving to the trial. To continue our research in this work, we studied the possibility of creating new water-soluble salts based on previously practically water-insoluble benzofuroxan derivatives that are of the great interest as potential anticancer agents and growth stimulators, and as regulators of various agricultural crops.

## 2. Results and Discussion

### 2.1. Chemistry

#### 2.1.1. Synthesis of the Water-Soluble Salts Based on Benzofuroxan

**Scheme 1:**
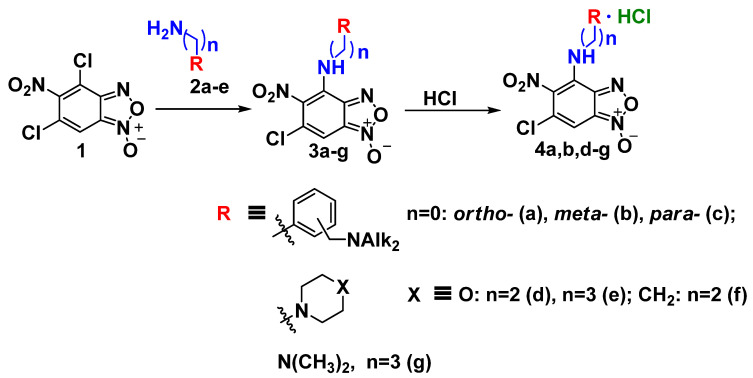
Obtaining water-soluble salts of benzofuroxans in two stages.

Key compounds were obtained via aromatic nucleophilic substitution reaction of 4,6-dichloro-5-nitrobenzofuroxan **1** using the aromatic amines **2a**–**e** as nucleophiles (Scheme 1).

It should be noted that in the case of compound **3c** no salt formation occurs due to the strong electron withdrawing effect of furoxan moiety in *para*-position.

**Figure 1:**
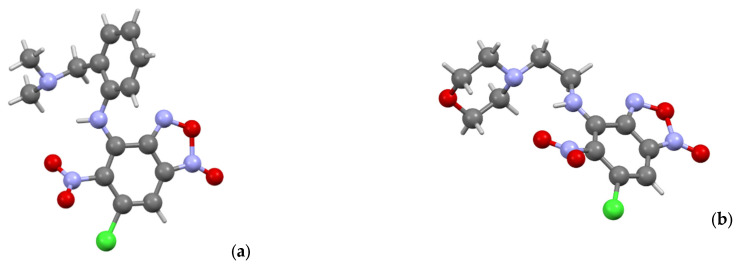

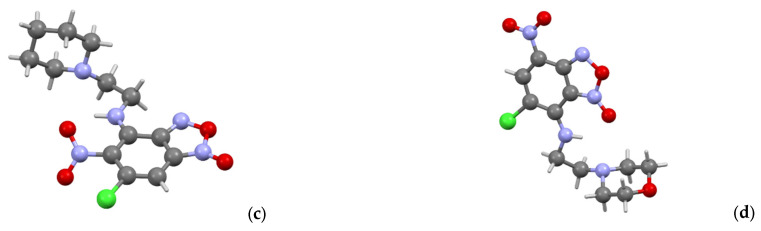
Molecular structures of compound **3a** (**a**), **3d** (**b**), **3f** (**c**) and **5d** (**d**).

In the case of the reaction of 4,6-dichloro-5-nitrobenzofuroxan with 2-morpholinoethanamine **2d**, in addition to the main reaction product **3d**, a by-product **5d** (Figure 1d) is formed in trace amounts (see the Section 2.1.2 for more details).

According to X-ray data (Figure 1, Tables S1 and S2, Supplementary Materials) compounds **3a**, **3d**, **3f** and **5d** crystallize in different space groups: compound **3a** crystallizes in the orthorhombic space group *P*2_1_2_1_2_1_, compounds **3d** and **5d** crystallize in the monoclinic space groups *P*2_1_/c and *P*2_1_/n, respectively, and compound **3f** crystallizes in the triclinic space group *P*-1. In this case, two independent molecules are present in the independent part of the unit cell of the crystal of compound **3f**. In all structures **3a**, **3d,3f**, and **5d**, the benzofuroxan fragments are planar. The nitro-groups in compounds **3a, 3d**, and **3f** are in the fifth position and deviate at sufficient angles from the plane of the benzene ring of the benzofuroxan fragment: in compound **3a**, the O^4^N^5^C^5^C^4^ torsion angle is 44.6(6)°; in compound **3d**, the O^9^N^8^C^5^C^4^ angle is 35.64(12)°; compound **3f** angle O^51A^N^5A^C^5A^C^4A^−35.13(17)° and O^51B^N^5B^C^5B^C^4B^−36.74(18)° for two independent molecules, respectively. In the crystal of compound **5d**, the nitro group is in the fourth position and lies almost in the plane of the benzene ring: the torsion angle O^9^N^8^C^4^C^3A^ is 5.8(8)°. The morpholine substituents in the crystals of compounds **3d** and **5d**, as well as the piperidine substituent in compound **3f**, have an “*chair*” conformation. The structures of these compounds in the crystal are stabilized by intramolecular hydrogen bonds involving the NH group of the substituent at the fourth position

#### 2.1.2. Quantum Chemical Calculations

**Scheme 2:**
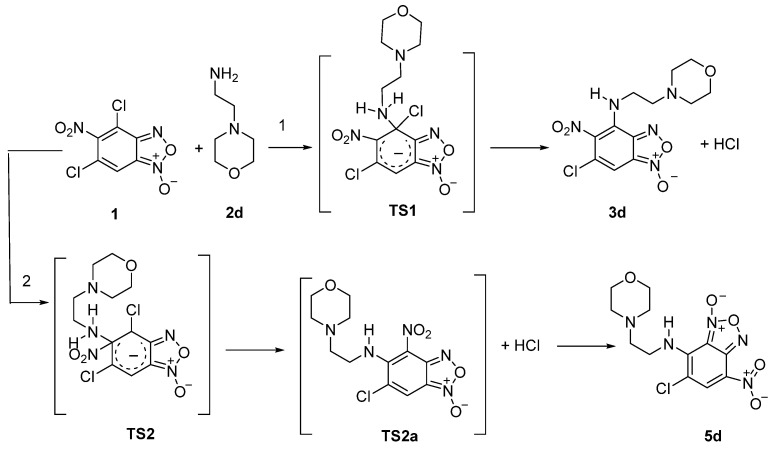
Two possible ways for the reaction of 4,6-dichloro-5-nitrobenzofuroxan (**1**) with 2-morpholinoethanamine (**2d**).

In order to evaluate the reaction mechanism of the 4,6-dichloro-5-nitrobenzofuroxan with 2-morpholinoethanamine **2d** (Scheme 2), we carried out a theoretical study of the reaction by methods of density functional theory using the B3LYP hybrid functional [29,30] in combination with the 6-31+G* basis set [31,32].

**Figure 2:**
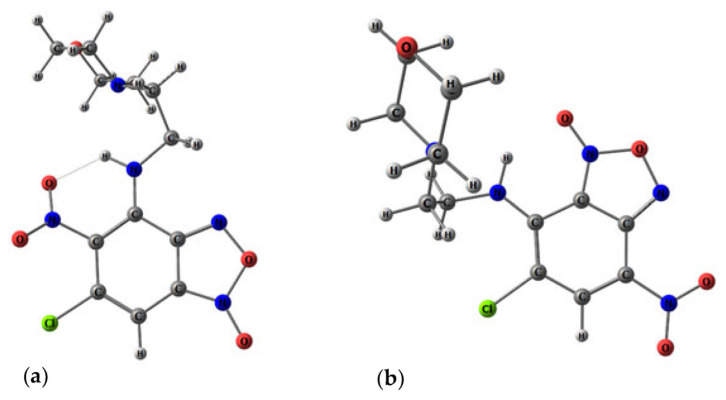
The structures of **3d** (**a**) **5d** (**b**).

According to the quantum chemical calculations results, the reaction of 4,6-dichloro-5-nitrobenzofuroxan **1** with 2-morpholinoethanamine **2d** is exothermic (thermal effect is 22.1 and 23.5 kcal/mol, respectively, for two expected reaction pathways, see Table 1) with **5d** being thermodynamically a little more stable compared to **3d**. Nevertheless, the higher HOMO-LUMO gap of **3d** suggests its more kinetical stability. The structures of **3d** and **5d** are presented in Figure 2.

**Figure 3:**
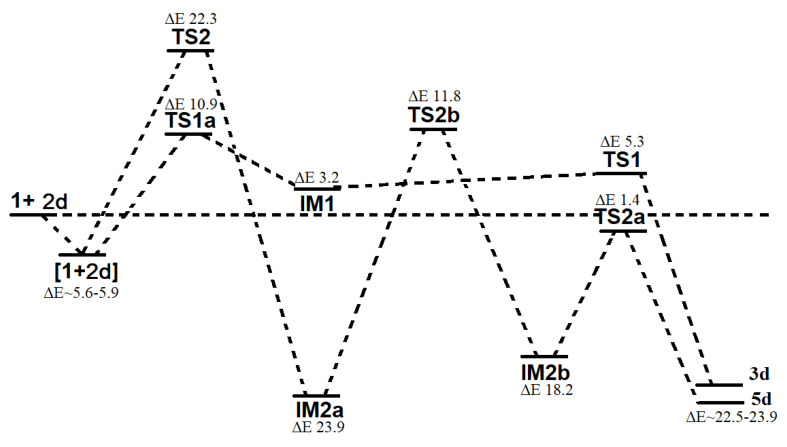
Energy diagram (ΔE, kcal/mol) of the reaction of 4,6-dichloro-5-nitrobenzofuroxan **1** with 2-morpholinoethanamine **2d**.

Thus, quantum chemical modeling shows comparable thermodynamic stabilities of products **3d** and **5d**. However, the higher reaction yield of the product **3d** indicates a significant difference in the reaction mechanisms. Calculations show that at the first stage pre-reaction complexes **[1+2d]a** and **[1+2d]b** are formed (ΔE ~6 kcal/mol, see Table 2), with the NH_2_-group of 2-morpholinoethanamine being located opposite to corresponding chlorine atom or N_2_O-group in benzofuroxan. Further, pre-reaction complexes form reaction products **3d** and **5d** through corresponding transition states and intermediates with activation energies of first transition states **TS1a** and **TS2** are 16.6 and 28.2 kcal/mol, respectively (see Table 2 and Figure 3, zero is sum of the total energies of reactants **1** and **2d** infinitely distant from each other), accompanied by the removal of hydrogen chloride.

**Scheme 3:**
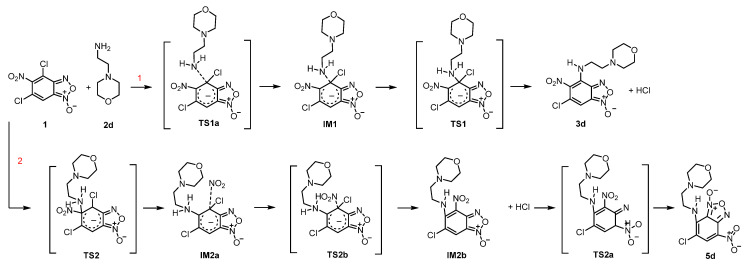
Two ways for the reaction of 4,6-dichloro-5-nitrobenzofuroxan **1** with 2-morpholinoethanamine **2d**.

Thus, two ways for the reaction of 4,6-dichloro-5-nitrobenzofuroxan **1** with 2-morpholinoethanamine **2d** are revealed by means of quantum-chemical investigations (Scheme 3). The results obtained fully confirm the experimental data and explain the higher reaction yield of the product **3d** in the reaction of 4,6-dichloro-5-nitrobenzofuroxan **1** with 2-morpholinoethanamine **2d** on the basis of its lower activation barrier.

### 2.2. Biological Studies

#### 2.2.1. Evaluation of Water-Soluble Salts Based on Benzofuroxanas Stimulants and Regulators of Crop Growth

The protection of plants from harmful organisms is of decisive importance in the cultivation of crops. The pre-sowing seed treatment is the main preventative measure in this case. More than 70% of infectious diseases are transmitted through the seed material [33]. To improve the sowing qualities of seeds, a wide range of stimulants and growth regulators is currently recommended (Fitolavin, Akpinol, Zircon) [34,35]. They improve seed germination, enhance plant growth and development, as well as plant resistance to diseases. At the same time, the saprophytic and pathogenic microflora of the seeds is suppressed, their germination energy increases, root formation, growth and the development of plants increase, and resistance to diseases during the growing season increases. The crop yield increases up to 30% in comparison with the control [36,37,38]. However, quite commonly used crop regulators and growth promoters are toxic and detrimental to the environment, as well as having a negative impact on human health. The development of low-toxic or non-toxic active substances, and having the above properties, is currently one of the urgent tasks we face. The water-soluble salts obtained by us based on benzofuroxans were evaluated as growth stimulators for various agricultural crops. To do this, in laboratory conditions, the effectiveness of the pre-sowing treatment of seeds of agricultural crops (wheat, alfalfa, barley, rice, sorghum) was evaluated. The results of experiments to assess the effect of compounds on the sowing qualities of seeds are presented in Table 3.

**Figure 4:**
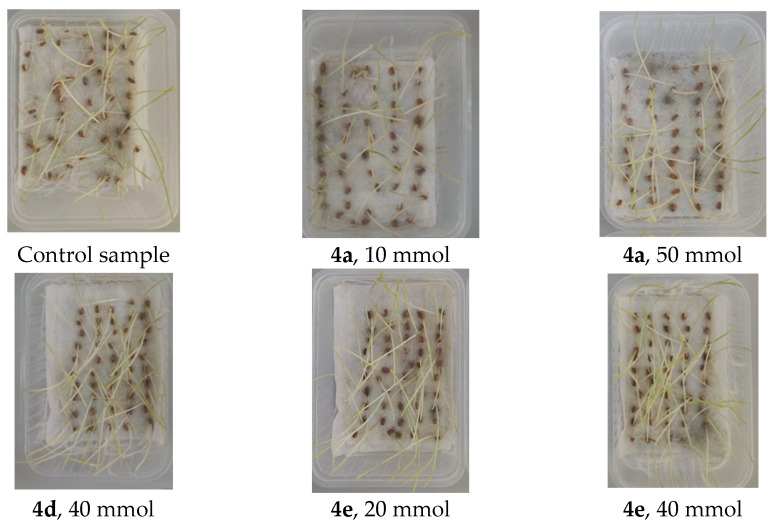
Sowing qualities of wheat seeds.

The results of the analysis showed that the sowing qualities of wheat seeds in the variant with the treatment of **4a**, 10 mmol and 50 mmol; **4d**, 40 mmol and **4e**, 20 mmol and 40 mmol, are higher than in the case of controls (Figure 4, lines 1–3 in gray, Table 3.

**Figure 5:**
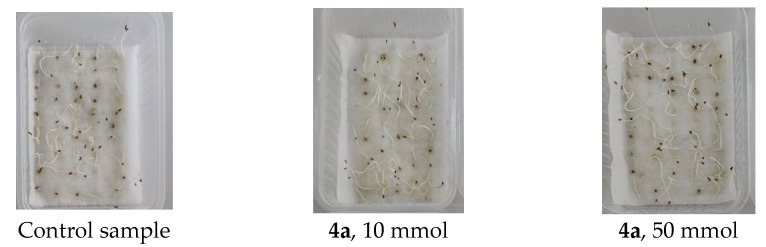
Sowing qualities of alfalfa seeds.

**Figure 6:**
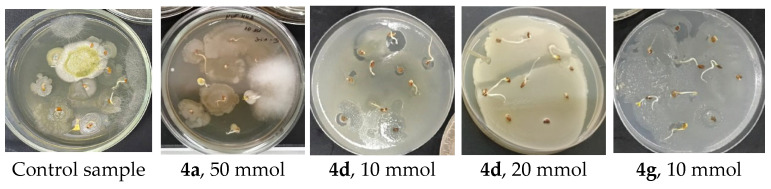
Growth of microflora on alfalfa seeds treated with compounds.

In the case of alfalfa seed treatment, sowing qualities in variants with **4a** treatment, 10 mmol and 50 mmol; **4g,** 40 mmol are higher than controls (Figure 5). Treatment with compounds **4a** at doses of 10 and 50 mmol; **4d**, 10 and 20 mmol and **4g**, 10 mmol, has a positive effect on the suppression of fungal microflora (Figure 6, line 9, Table 3).

**Figure 7:**
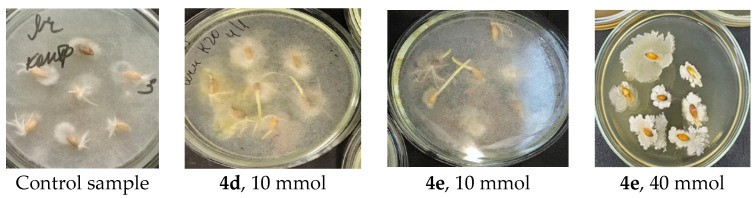
Growth of microflora on barley seeds treated with compounds.

When processing barley seeds, it was shown that the sowing qualities of seeds treated with compounds **4a**, 10 and 50 mmol; **4e**, 20 mmol and **4g**, 20 and 40 mmol, are higher than control (lines 11–13 in gray, Table 3). A slight suppression of the bacterial microflora was noted in the treatment with compounds **4d** at doses of 10 and 40 mmol and **4e** at all studied doses (Figure 7, line 15, Table 3).

Rice seed treatment in variants with compounds **4a**, 10 and 50 mmol; **4d**, 40 mmol; **4e**, 20 and 40 mmol (lines 16–18 in gray, Table 3) showed better results than the control, and the variants with compounds **4g**, 40 mmol were at the control level. Treatment with compounds **4d** and **4e** at low doses of 10 and 20 mmol, has a positive effect on the suppression of bacterial microflora (line 20, Table 3).

In the case of sorghum seed treatment, the results of the experiment showed that the sowing qualities of seeds treated with compounds **4d**, 20 mmol and **4g**, 20 and 40 mmol are higher than in the control (lines 21–23 in grey, Table 3). The variant with compound **4a**, 50 mmol was at the control level.

Thus, the studied substances at concentrations of 20–40 mmol show good effectiveness of the pre-sowing treatment of seeds of agricultural crops. It should be noted that the introduction of an aromatic amine moiety improves the properties, and compound **4a** shows activity already at a concentration of 10 mmol. It should also be noted that in some cases, seed treatment with water-soluble salts of benzofuroxans leads not only to improved seed germination, but also to the suppression of microflora growth.

#### 2.2.2. Anticancer Activity

##### Cytotoxicity of the Test Compounds

Currently, cancer is one of the most serious problems facing humanity, requiring the development of new drugs and treatment methods. Cases of cancer therapy methods improving, leading to a sharp enhancement intreatment outcomes, are extremely rare, and in general most of the drugs used to treatcancer are toxic to the body [39]. Most of the used anticancer agents have poor water solubility, which adversely affects their efficacy and safety when administered orally, intramuscularly, and intravenously. The poor aqueous solubility of anticancer drugs results in poor efficacy or requires the use of excipients that have toxic side effects. Attempts have been made to solve the problem of poor aqueous solubility of antitumor drugs, such as the use of prodrugs, polymeric nanoparticles, lipoid microspheres, solubilizers, and nanocolloids [40,41,42,43,44,45,46]. However, these attempts are limited by a number of problems such as instability, the potential toxicity of the compounds, the altered distribution of the drug in the body, and so on [47,48]. As described earlier, the main emphasis in the synthetic part was made precisely on obtaining the effective water-soluble salts of benzofuroxans. All synthesized compounds were tested in order to find promising agents with antitumor potential.

In the classical script in the search for promising compounds with a potential antitumor effect, substances that exhibit a cytotoxic effect on tumor-derived cells are primarily of interest. This is due to the fact that the main focus of chemotherapeutic agents is to suppress the already formed malignant neoplasms viability, and prevent the formation of new ones.

In this work, the cytotoxic potential of the water-soluble salts based on benzofuroxan was determined by the effect of compounds on the survival of the cell tumor origin cultures (SH-SY5Y, Hep2, A549, HeLa) using the MTT test. To understand how the synthesized compounds can influence the healthy microenvironment in the body, the effect of substances on the viability of a normal Hek 293 cell culture was additionally evaluated.

As shown in Table 4, at 24 h of incubation, all test compounds showed cytotoxic properties, especially in relation to SH-SY5Y. The most pronounced effect on the majority of used cell lines of tumor origin was observed for compounds **4e** and **4g**. At the same time, for compound **4g**, high toxicity was also observed in relation to normal cells. In turn, for **4e**, the selectivity index reached a value of more than 4 when comparing the IC_50_ on the neuroblastoma SH-SY5Y with respect to Hek 293.

Additionally, we carried out a comparative analysis of the survival of cell cultures during incubation with water-soluble salts based on benzofuroxan for two time periods—24 and 72 h. It was found that with an increase in the duration of exposure to substances with selected cell lines, the values of concentrations that cause 50% inhibition of the cell population decrease growth. This may be due to both an increase in the direct toxic effect of the synthesized compounds over time, and the presence of possible antiproliferative activity, which is expressed in the effect of substances on the cell division process, since several cell cycles occur within 72 h.

Metabolic transformation is involved in various processes of malignant neoplasms formation and progression, including uncontrolled proliferation and the resistance of tumor cells to apoptosis [49,50]. To ask whether the effect of water-soluble salts based on benzofuroxan on the reduction in cell survival is due to their effect on metabolism, the ability of the compounds to modulate glycolysis, the process that provides neoplastic cells with ATP and building blocks for the synthesis of macromolecules, was measured.

**Figure 8:**
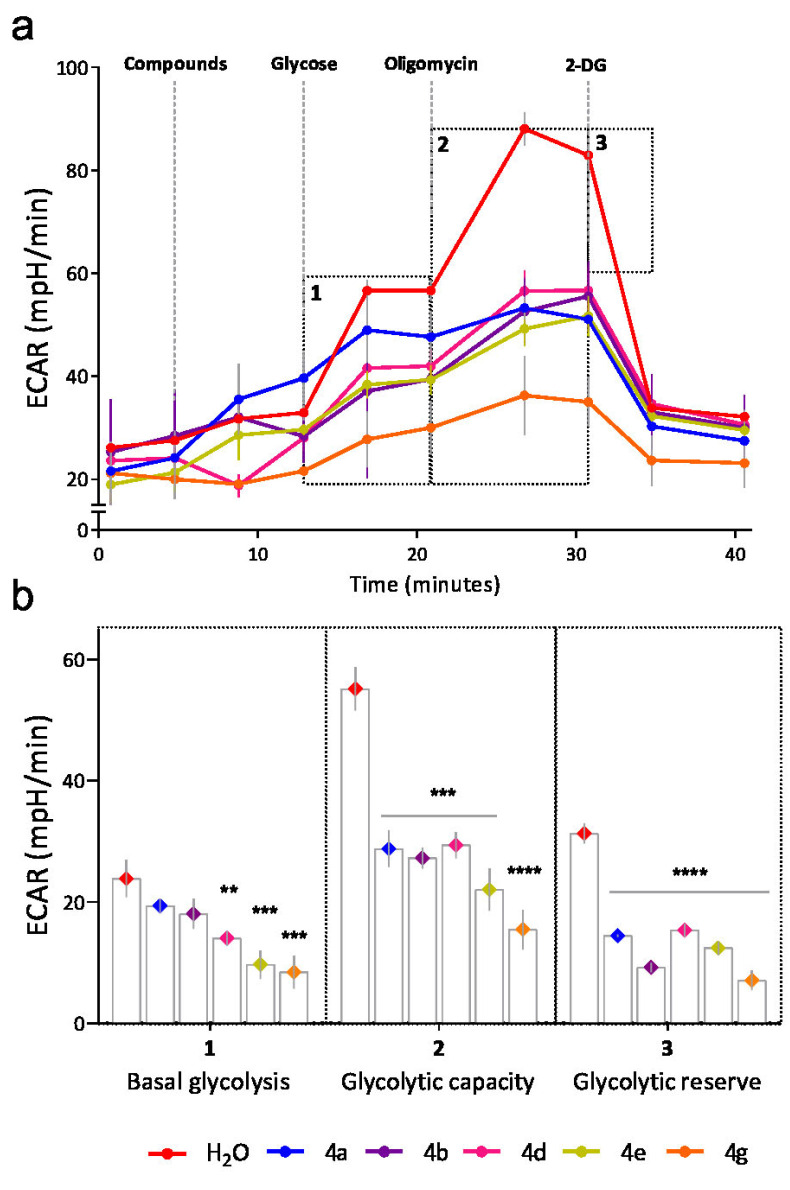
The influence of the studied compounds on (**a**) the glycolytic profile of tumor origin cells SH-SY5Y (neuroblastoma) and (**b**) parameters of glycolytic function. The extracellular acidification rate (ECAR) is a glycolysis indicator and shows the degree of extracellular environment acidification. The concentration of the studied compounds was 100 µM, glucose was 10 mM, oligomycin was 1 µM, and 2-fluoro-2-deoxy-D-glucose was 25 mM. Data are presented as mean ± error of the mean (n = 3). ** *p* ≤ 0.01, *** *p* ≤ 0.001, **** *p* ≤ 0.0001 compared to control (one-way ANOVA).

Using the cell metabolism analyzer Agilent Seahorse XF96e Analyzer (Seahorse Bioscience, North Billerica, MA, USA), the rate of extracellular acidification of the medium (ECAR) in cells of tumor origin SH-SY5Y was evaluated as a glycolysis measure. The effect of compounds on glycolytic function parameters was analyzed by monitoring changes in ECAR in response to sequential addition of glucose to evaluate glycolysis and oligomycin (Figure 8a). Interestingly, all glycolytic parameters were significantly reduced in SH-SY5Y cells treated with 100 µM of most water-soluble salts based on benzofuroxan, including basal glycolysis, glycolytic capacity, and glycolytic reserve, reaching a minimum after **4g** injection. Moreover, cells treated with **4e** also showed a significantly more pronounced suppression of glycolytic function parameters—by 60%—when compared with control samples (Figure 8b).

Together, these data indicate that **4e** and **4g** treatment of the tumor-derived cells has a strong influence on the glycolytic energy pathway of the tumor cells.

Thus, the compounds containing morpholine fragments, as well as a fragment of *N*-dimethylpropylamine **4e** and **4g** demonstrated the highest activity and were selected for the further experiments using laboratory animals.

##### Acute Toxicity

**Figure 9:**
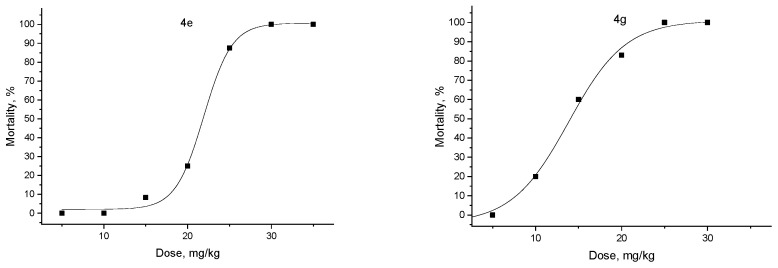
Dose-response curves for single intraperitoneal administration of **4e** and **4g** to BDF_1_ hybrid mice.

To determine LD_50_, a curve of increasing toxicity was built with increasing dose, based on which the following parameters were additionally determined: MTD, LD_16_, LD_84_ and LD_100_. Compounds **4e** and **4g** were intraperitoneally administered to BDF_1_ mice once in the dose range of 10–35 mg/kg and 5–25 mg/kg, respectively. The results are presented in the Figure 9 and Table 5.

It was shown that the death of animals began within 5–30min after the administration of the studied compounds **4e** and **4g** to mice at doses of 15–30 mg/kg and 10–25 mg/kg, respectively. At the same time, excitability increased in animals, convulsions were observed, after which the death of experimental animals occurred. In surviving mice, no other external signs of intoxication were observed. We did not record pathological changes in the eyes (lacrimation, swelling, hyperemia, etc.), and the nose, ears, and teeth did not differ from those in control animals. Visible mucous membranes are normal. The skin is unchanged. The volume and consistency of fecal matter, and the color of urine is also normal. During the subsequent observation period, the general condition and behavior of the surviving animals of all experimental groups corresponded to the usual. There were no differences in these parameters between the experimental and control groups.

Data on changes in the body weight of experimental animals under the action of **4e** and **4g** are presented in Table 6 and Table 7, respectively. It was shown that after a single administration of the test compounds, the average body weight of surviving experimental animals did not significantly change relative to the control group throughout the entire observation period.

Thus, it was found that with a single intraperitoneal injection of compound **4e** in the dose range of 15–30 mg/kg and **4g** in the dose range of 10–25 mg/kg, the death of animals occurs within half an hour after the administration of the test compounds. The values of LD_50_=22.0 ± 1.33 mg/kg for **4e** and LD_50_ = 13.75 ± 1.73 mg/kg for **4g** were determined. The results of the study of acute toxicity make it possible to attribute **4e** and **4g** to the class of highly toxic compounds.

##### Antitumor Activity In Vivo

**Figure 10:**
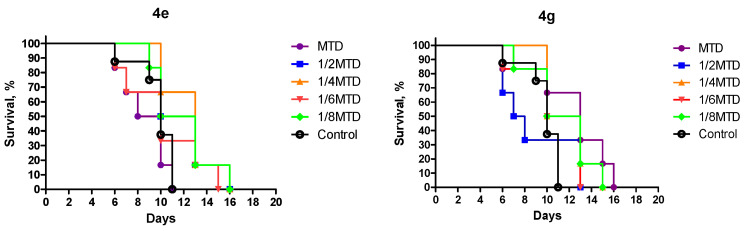
Survival curves of BDF_1_ mice with leukemia P388 after a single intraperitoneal injection of compounds **4e** and **4g**.

The study of the antitumor activity of compounds **4e** and **4g** was carried out using transplanted experimental murine tumor leukemia P388. In the experiment, concentrations from 1.25 to 10 mg/kg were used for **4e**, and from 0.625 to 5 mg/kg for **4g**. The concentrations used correspond to values from 1/8 MPD to MPD for the studied compounds. The results are shown in the Figure 10.

It was shown that in the control group all the studied animals die by day 11, while the administration of the benzofuroxan derivatives **4e** and **4g** at doses from 1/8 MPD to MPD led to an increase in the lifespan of animals up to 16 days.

It was found that chemotherapy of P388 leukemia using a single injection of **4e** at a dose of 2.5 mg/kg leads to an increase in the average lifespan of animals by 28%. Similarly, when using **4g** at a dose of 5 mg/kg, a 25% increase in average lifespan was observed. It should also be noted that even using a dose of 1.25 mg/kg resulted in a ~20% increase in ILS% for both test substances.

Thus, water-soluble salts of benzofuroxans can be used as a promising basis for the development of effective antitumor agents.

## 3. Materials and Methods

### 3.1. Chemistry

IR spectra were recorded on an IR Fourier spectrometer Tensor 37 (Bruker Optik GmbH, Germany) in the 400–3600 cm^−1^ range in KBr. The ^1^H- and ^13^C-NMR spectra were recorded on a Bruker AVANCE 400 spectrometer (Bruker BioSpin, Rheinstetten, Germany) operating at 400 MHz (for ^1^H NMR) and 101 MHz (for ^13^C NMR), Brucker spectrometers AVANCE*III*-500 (Bruker BioSpin, Rheinstetten, Germany) operating at 500 MHz (for ^1^H NMR) and 126 MHz (for ^13^C MMR) and Bruker Avance 600 spectrometer (Bruker BioSpin, Rheinstetten, Germany) operating at 600 MHz (for ^1^H NMR) and 151 MHz (for ^13^C NMR) (Figures S1–S23, Supplementary Materials). Chemical shifts were measured in δ (ppm) with reference to the solvent (δ = 7.27 ppm and 77.00 ppm for CDCl_3_, δ = 2.06 ppm and 28.94 ppm for (CD_3_)_2_CO, δ = 4.79 ppm for D_2_O for ^1^H and ^13^C NMR, respectively). Elemental analysis was performed on a CHNS-O Elemental Analyser EuroEA3028-HT-OM (EuroVector S.p.A., Milan, Italy). The melting points were determined on JK-MAM-4 Melting-point Apparatus with Microscope (JINGKE SCIENTIFIC INSTRUMENT CO, Shanghai, China).

**X-ray crystallography data.** Data of **3a**, **3d**, **3f** and **5d** were collected on a Bruker D8 QUEST with PHOTON II CCD diffractometer (Bruker AXS, Ettlingen, Germany), using graphite monochromatedMoKα (λ = 0.71073 Å) radiation and ω-scan rotation. Data collection images were indexed, integrated, and scaled using the APEX3 [51] data reduction package^70^ and corrected for absorption using SADABS [52]. The structure was solved by direct methods and refined using SHELX program [53]. All non-hydrogen atoms were refined anisotropically. H atoms were calculated on idealized positions and refined as riding atoms. Crystal Data and Refinement Details are presented in Tables S1 and S2. The X-ray analysis was performed on the equipment of Spectral-Analytical Center of FRC Kazan Scientific Center of RAS.

CCDC 2220082–2220085 (**3a**, **3d**, **3f** and **5d**) contain the supplementary crystallographic data for this paper. These data can be obtained free of charge via www.ccdc.cam.ac.uk/conts/retrieving.html (accessed on 26 October 2022) or from the Cambridge Crystallographic Data Centre, 12 Union Road, Cambridge CB2 1EZ, UK; fax: (+44) 1223-336-033; or deposit@ccdc.cam.uk).

**Quantum-chemical computations.** All quantum-chemical computations were carried out with the use of Gaussian 16 suite of programs [54]. Calculations were performed with Becke’s three parameter hybrid exchange functional [29] and the gradient-corrected nonlocal correlation functional of Lee et al. [30] (B3LYP) in combination with standard 6–31+G* basis set [31,32,55]. For all compounds, geometry optimization of structures was performed without symmetry constraints. To ensure the calculated structures of reagents and products were indeed minima, vibrational analyses were performed using the same methods and were proved by all positive eigenvalues of Hessian matrix. The transition states were confirmed by the presence of one negative eigenvalue in the Hessian matrix of the second derivatives. Additionally, intrinsic reaction coordinate (IRC) was performed to examine connections between all the species involved in the reaction. All calculations were performed for a singlet surface and the solutions found were tested for stability against perturbations imposed on the wave function using the Stable procedure.

The completeness of the reactions and the purity of the synthesized compounds were monitored by thin layer chromatography (TLC) on Sorbfil PTSH-AF-A-UF plates (Sorbpolimer, Krasnodar, Russia), UV light was used as a developer.

The following compounds were prepared following the literature procedures indicated: 4,6-dichloro-5-nitrobenzofuroxan **1** [56], 6-chloro-4-(3-morpholinoprophylamino)-5-nitrobenzo[*c*][1,2,5]oxadiazole 1-oxide **3e** [28].

**Synthesis of amine-containing benzofuroxans 3a-f (general method).** A solution of amine (1.6 mmol) in chloroform (2 mL) was added to a solution of benzofuroxan**1** (0.8 mmol) in chloroform (2 mL) at room temperature with stirring. The reaction mixture was kept for 2 h at room temperature with constant stirring (the reaction progress and the purity of the obtained products were monitored by TLC, eluent toluene:ethyl acetate, 2:1). At the end of the exposure, the reaction mixture was reprecipitated in hexane (10 mL), the resulting precipitate was filtered off, washed with water (100 mL), and dried in vacuum (0.06 mm Hg) at a temperature of 40 °C to constant weight.

**6-chloro-4-(2-((dimethylamino)methyl)phenylamino)-5-nitrobenzo[*c*][1,2,5]oxadiazole 1-oxide (3a)**. Brick red powder. Yield 276 mg (95%), m.p. = 152–153 °C with decomposition. IR spectrum, ν, cm^−1^: 721 (CCl), 1313 (NO_2_ symm), 1563 (NO_2_ asymm), 1624 (furoxan ring), 3084 (C^7^H).^1^H NMR (400 MHz, CDCl_3_) δ 7.19 (m, 2H, Ph), 7.13 (m, 1H, Ph), 7.05 (d, *J* = 7.9 Hz, 1H, Ph), 6.90 (s, 1H, Bf), 3.63 (s, 2H, CH_2_), 2.32 (s, 6H, CH_3_). ^13^C NMR (101 MHz, CDCl_3_) δ 147.3, 139.4, 134.8, 130.4, 129.3, 128.7, 128.2, 125.4, 121.3, 114.0, 106.7, 102.1, 63.4, 44.9. Found: C, 49.57; H, 3.89; Cl, 9.72; N, 19.21. Anal. calcd (%) for C_15_H_14_ClN_5_O_4_: C, 49.53; H, 3.88; Cl, 9.75; N, 19.25.

**6-chloro-4-(3-((dimethylamino)methyl)phenylamino)-5-nitrobenzo[*c*][1,2,5]oxadiazole 1-oxide (3b)**. Purple powder. Yield 220 mg (76%), m.p. = 118–119 °C. IR spectrum, ν, cm^−1^: 700 (CCl), 1310 (NO_2_ symm), 1561 (NO_2_ asymm), 1625 (furoxan ring).^1^H NMR (400 MHz, acetone-d_6_) δ 7.31 (m, 2H, Ph), 7.23 (m, 2H, Ph), 7.13 (s, 1H, Bf), 3.40 (s, 2H, CH_2_), 2.19 (s, 6H, CH_3_). ^13^C NMR (101 MHz, acetone-d_6_) δ 148.2, 141.0, 139.1, 133.3, 131.9, 129.2, 127.9, 127.4, 124.9, 123.2, 114.1, 102.5, 64.0, 45.2. Found: C, 49.55; H, 3.86; Cl, 9.78; N, 19.22. Anal. calcd (%) for C_15_H_14_ClN_5_O_4_: C, 49.53; H, 3.88; Cl, 9.75; N, 19.25.

**6-chloro-4-(4-((dimethylamino)methyl)phenylamino)-5-nitrobenzo[*c*][1,2,5]oxadiazole 1-oxide (3c)**. Dark brown powder. Yield 247 mg (85%), m.p. > 300 °C with decomposition. IR spectrum, ν, cm^−1^: 722 (CCl), 1536 (NO_2_ asymm), 1612 (furoxan ring).^1^H NMR (400 MHz, CDCl_3_) δ 9.01 (s, 1H, NH), 7.39 (d, *J* = 8.4 Hz, 2H, Ph), 7.35 (s, 1H, Bf), 7.21 (d, *J* = 8.4 Hz, 2H, Ph), 3.52 (s, 2H, CH_2_), 2.30 (s, 6H, CH_3_). ^13^C NMR (101 MHz, CDCl_3_) δ 148.8, 144.2, 139.2, 137.3, 134.1, 132.4, 130.7, 130.4, 125.6, 106.5, 64.2, 45.8. Found: C, 49.58; H, 3.84; Cl, 9.74; N, 19.28. Anal. calcd (%) for C_15_H_14_ClN_5_O_4_: C, 49.53; H, 3.88; Cl, 9.75; N, 19.25.

**6-chloro-4-(2-morpholinoethylamino)-5-nitrobenzo[*c*][1,2,5]oxadiazole 1-oxide (3d)**. Bright orange powder. Yield 266 mg (98%), m.p. = 140–141 °C. IR spectrum, ν, cm^−1^: 679 (CCl), 1387 (NO_2_ symm), 1579 (NO_2_ asymm), 1627 (furoxan ring), 3052 (C^7^H). ^1^H NMR (400 MHz, acetone-d_6_) δ 8.87 (s, 1H, NH), 6.79 (s, 1H, Bf), 4.19 (d, *J* = 5.6 Hz, 2H, CH_2_), 3.67 (s, 4H, CH_2_), 2.80 (s, 4H, CH_2_), 2.57 (m, 2H, CH_2_). ^13^C NMR (101 MHz, acetone-d_6_, δ, m.d.): 148.4, 138.2, 128.9, 127.3, 113.0, 98.8, 66.6, 55.9, 52.9, 43.1. Found: C, 41.96; H, 4.09; Cl, 10.33; N, 20.34. Anal. calcd (%) for C_12_H_14_ClN_5_O_5_: C, 41.93; H, 4.11; Cl, 10.31; N, 20.37.

**6-chloro-5-nitro-4-(2-(piperidin-1-yl)ethylamino)benzo[*c*][1,2,5]oxadiazole 1-oxide (3f)**. Purple powder. Yield 205 mg (75%), m.p. = 88–89 °C. IR spectrum, ν, cm^−1^: 687 (CCl), 1366 (NO_2_ symm), 1561 (NO_2_ asymm), 1627 (furoxan ring), 3091 (C^7^H). ^1^H NMR (600 MHz, acetone-d_6_, δ): 8.92 (s, 1H, NH), 6.77 (s, 1H, Bf), 4.14 (m, 2H, CH_2_), 2.73 (m, 2H, CH_2_), 2.51 (m, 4H, CH_2_), 1.61 (m, 4H, CH_2_), 1.49 (m, 2H, CH_2_). ^13^C NMR (151 MHz, acetone-d_6_) δ 148.5, 138.1, 129.1, 127.2, 113.1, 98.6, 56.0, 53.7, 43.5, 26.0, 24.2. Found: C, 45.72; H, 4.70; Cl, 10.34; N, 20.45. Anal. calcd (%) for C_13_H_16_ClN_5_O_4_: C, 45.69; H, 4.72; Cl, 10.37; N, 20.49.

**6-chloro-4-(3-(dimethylamino)propylamino)-5-nitrobenzo[*c*][1,2,5]oxadiazole 1-oxide (3g).** Orange powder. Yield 219 mg (87%), m.p.= 55–56 °C. IR spectrum, ν, cm^−1^: 684 (CCl), 1348 (NO_2_ symm), 1536 (NO_2_ asymm), 1619 (furoxan ring), 3104 (C^7^H), 3372 (NH).^1^H NMR (400 MHz, CDCl_3_) δ 10.01 (m, 1H, NH), 6.56 (s, 1H, Bf), 4.14 (m, 2H, CH_2_), 2.57 (m, 6H, CH_3_), 2.32 (s, 2H, CH_2_), 1.87 (m, 2H, CH_2_). ^13^C NMR (101 MHz, CDCl_3_) δ 147.9, 136.6, 129.6, 125.5, 113.2, 97.4, 58.7, 48.3, 45.0, 25.0. Found: C, 41.80; H, 4.44; Cl, 11.28; N, 22.11. Anal. calcd (%) for C_11_H_14_ClN_5_O_4_: C, 41.85; H, 4.47; Cl, 11.23; N, 22.18.

**Synthesis of benzofuroxans salts 4a-f (general method).** Hydrochloric acid (1.0 mmol) was added dropwise to a solution of benzofuroxan**3a-f** (0.8 mmol) in ethyl alcohol (2 mL) at room temperature with stirring. The reaction mixture was kept overnight at room temperature with constant stirring. At the end of the exposure, the precipitate that formed was filtered off and dried in a vacuum (0.06 mm Hg) at a temperature of 40 °C to constant weight.

**6-chloro-4-(2-((dimethylammonio)methyl)phenylamino)-5-nitrobenzo[*c*][1,2,5]oxadiazole 1-oxide chloride (4a).** Orange powder. Yield 260 mg (82%), m.p. = 125–126 °C with decomposition. IR spectrum, ν, cm^−1^: 689 (CCl), 1348 (NO_2_ symm), 1555 (NO_2_ asymm), 1623 (furoxan ring), 2465 (NH^+^), 3100 (C^7^H).^1^H NMR (600 MHz, D_2_O) δ 7.70 (d, *J* = 7.3 Hz, 1H, Ph), 7.55 (m, 2H, Ph), 7.36 (d, *J* = 7.6 Hz, 1H, Ph), 7.04 (s, 1H, Bf), 4.52 (s, 2H, CH_2_), 2.96 (s, 6H, CH_3_). ^13^C NMR (101 MHz, D_2_O) δ 147.9, 138.4, 133.1, 132.8, 132.4, 131.8, 129.6, 128.7, 128.3, 127.2, 114.9, 102.7, 57.2, 43.3. Found: C, 44.96; H, 3.81; Cl, 17.79; N, 17.73. Anal. calcd (%) for C_15_H_15_Cl_2_N_5_O_4_: C, 45.02; H, 3.78; Cl, 17.72; N, 17.50.

**6-chloro-4-(3-((dimethylammonio)methyl)phenylamino)-5-nitrobenzo[*c*][1,2,5]oxadiazole 1-oxide chloride (4b).** Orange oil. Yield 310 mg (96%). IR spectrum, ν, cm^−1^: 699 (CCl), 1354 (NO_2_ symm), 1563 (NO_2_ asymm), 1624 (furoxan ring), 2478 (NH^+^), 3407 (NH).^1^H NMR (600 MHz, D_2_O) δ 7.55 (m, 3H, Ph), 7.48 (s, 1H, Ph), 7.46 (s, 1H, Bf), 4.31 (s, 2H, CH_2_), 2.79 (s, 6H, CH_3_). ^13^C NMR (101 MHz, D_2_O) δ 143.7, 132.7, 132.0, 131.9, 131.8, 131.7, 131.3, 125.8, 125.2, 124.2, 114.0, 96.9, 60.6, 42.8. Found: C, 45.01; H, 3.85; Cl, 17.81; N, 17.56. Anal. calcd (%) for C_15_H_15_Cl_2_N_5_O_4_: C, 45.02; H, 3.78; Cl, 17.72; N, 17.50

**6-chloro-4-(2-(morpholino-4-ium)ethylamino)-5-nitrobenzo[*c*][1,2,5]oxadiazole 1-oxide chloride (4d).** Orange powder. Yield 230 mg (76%), m.p. = 186–187 °C. IR spectrum, ν, cm^−1^: 688 (CCl), 1392 (NO_2_ symm), 1577 (NO_2_ asymm), 1630 (furoxan ring), 2481 (NH^+^), 3248 (NH).^1^H NMR (400 MHz, D_2_O) δ 6.72 (s, 1H, Bf), 4.42 (t, *J* = 6.7 Hz, 2H, CH_2_), 4.02 (br.s, 4H, CH_2_), 3.59 (t, *J* = 6.7 Hz, 4H, CH_2_), 3.49 (br.s, 4H, CH_2_). ЯMP^13^C (101 Hz, D_2_O) δ 147.9, 137.1, 129.6, 129.0, 114.6, 100.5, 64.3, 56.6, 52.9, 40.7. Found: C, 37.98; H, 3.93; Cl, 18.67; N, 18.49. Anal. calcd (%) for C_12_H_15_Cl_2_N_5_O_5_: C, 37.91; H, 3.98; Cl, 18.65; N, 18.42.

**6-chloro-4-(3-(morpholino-4-ium)propylamino)-5-nitrobenzo[*c*][1,2,5]oxadiazole 1-oxide chloride (4e).** Red powder. Yield 250 mg (79%), m.p.= 239–240 °C. IR spectrum, ν, cm^−1^:1376 (NO_2_ symm), 1567 (NO_2_ asymm), 1613 (furoxan ring), 2361 (NH^+^), 3197 (NH).^1^H NMR (400 MHz, D_2_O) δ 6.88 (s, 1H, Bf), 4.12 (m, 4H, CH_2_), 3.85 (m, 2H, CH_2_), 3.53 (m, 2H, CH_2_), 3.28 (m, 4H, CH_2_), 2.24 (m, 2H, CH_2_). ^13^C NMR (101 MHz, D_2_O) δ 147.9, 138.4, 129.8, 126.3, 114.7, 99.9, 63.9, 54.5, 51.9, 43.3, 24.3. Found: C, 39.68; H, 4.32; Cl, 17.95; N, 17.82. Anal. calcd (%) for C_13_H_17_Cl_2_N_5_O_5_: C, 39.61; H, 4.35; Cl, 17.99; N, 17.77.

**6-chloro-5-nitro-4-(2-(piperidinium-1-yl)ethylamino)benzo[*c*][1,2,5]oxadiazole 1-oxide chloride (4f).** Red powder. Yield 230 mg (78%), m.p. = 106–107 °C. IR spectrum, ν, cm^−1^:697 (CCl), 1373 (NO_2_ symm), 1561 (NO_2_ asymm), 1613 (furoxan ring), 2530 (NH^+^), 3099 (C^7^H), 3429 (NH). ^1^H NMR (500 MHz, D_2_O) δ 6.92 (s, 1H, Bf), 4.39 (m, 2H, CH_2_), 3.60 (d, *J* = 11.9 Hz, 2H, CH_2_), 3.48 (m, 2H, CH_2_), 3.03 (t, *J* = 12.1 Hz, 2H, CH_2_), 1.94 (m, 2H, CH_2_), 1.76 (m, 4H, CH_2_). ^13^C NMR (126 MHz, D_2_O) δ 147.7, 137.1, 129.3, 128.7, 114.5, 100.5, 55.64, 53.8, 40.3, 22.6, 20.9. Found: C, 41.17; H, 4.57; Cl, 18.82; N, 18.51. Anal. calcd (%) for C_13_H_17_Cl_2_N_5_O_4_: C, 41.28; H, 4.53; Cl, 18.75; N, 18.52.

**6-chloro-4-(3-(dimethylammonio)propylamino)-5-nitrobenzo[*c*][1,2,5]oxadiazole 1-oxide chloride (4g).** Orange powder. Yield 200 mg (73%), m.p. = 196–197 °C with decomposition. IR spectrum, ν, cm^−1^: 687 (CCl), 1370 (NO_2_ symm), 1566 (NO_2_ asymm), 1621 (furoxan ring), 2481 (NH^+^), 3100 (C^7^H),3342 (NH).^1^H NMR (400 MHz, D_2_O) δ 6.57 (m, 1H, Bf), 4.13 (t, *J* = 6.9 Hz, 2H, CH_2_), 3.39 (m, 2H, CH_2_), 3.03 (s, 6H, CH_3_), 2.30 (m, 2H, CH_2_). ^13^C NMR (101 MHz, D_2_O) δ 147.9, 138.1, 129.8, 127.5, 114.3, 99.4, 55.5, 44.0, 43.5, 25.7. Found: C, 37.62; H, 4.33; Cl, 20.25; N, 19.93. Anal. calcd (%) for C_11_H_15_Cl_2_N_5_O_4_: C, 37.51; H, 4.29; Cl, 20.13; N, 19.89.

### 3.2. Biological Studies

#### 3.2.1. Evaluation of Water-Soluble Salts Based on Benzofuroxanas Stimulants and Regulators of Crop Growth

During phytopathological analyses of agricultural crops, the species composition of fungal and bacterial microflora was established. Laboratory studies were carried out on two nutrient media potato-agar (ingredients: potatoes—200 g; agar-agar—20 g; distilled water—1000 mL.) and potato-dextrose agar (ingredients: potatoes—200 g;dextrose—20 g; agar-agar—20 g; distilled water—1000 mL), according to the guidelines [57,58,59].

When evaluating the effectiveness of treatment of wheat, alfalfa, barley, rice, and sorghum seeds as fungicidal and bactericidal agents with preparation compounds at concentrations of 10, 20, and 40 mmol, their effect on the suppression of seed infection was evaluated. At the same time, it was noted that these drugs do not suppress fungal and bacterial infection, but stimulate the growth of seedlings in comparison with the control.

In laboratory conditions, the effectiveness of the preparation compounds were evaluated at a concentration of 10, 20, and 40 mmol on seeds of wheat, alfalfa, barley, rice, and sorghum. The sowing qualities were evaluated (germination energy for 3–4 days, laboratory germination for 7–8 days) according to GOST 12038-84 [60]. Sowing qualities of seeds were determined in wet chambers. According to the sample, 50 seeds were taken in 4-fold repetition.

#### 3.2.2. Cell Lines and Their Cultivation

Human cell cultures of tumor origin—SH-SY5Y (neuroblastoma), HeLa (cervical tumor), Hep-2 (larynx carcinoma), A549 (adenocarcinomic human alveolar basal epithelial cells)—and normal cell line Hek 293 (human embryonic kidneys), provided by the Laboratory of Tumor Cell Genetics of the Scientific Research Institute of Carcinogenesis, N.N. Blokhin National Medical Research Center of Oncology, as well as the Institute of Cytology of the Russian Academy of Sciences, were grown in a nutrient medium DMEM (PanEco, Moscow, Russia) and MEM (PanEco, Moscow, Russia), containing fetal bovine serum (10% by volume) (ThermoFisher Scientific, Paisley, UK), Glutamax (2 mM) (Gibco, Scotland, UK), and penicillin-streptomycin (1% by volume) (PanEco, Moscow, Russia). The cultivation was carried out at 37 °C in a humidified CO_2_ atmosphere (5%).

#### 3.2.3. Determination of Cell Viability

Cell viability was determined by MTT test [61]. Cells were seeded in a 96-well plate in the amount of 1 × 10^4^ cells/200µLof complete nutrient medium and cultured at 37 °C in CO_2_ (5%). After 24 h of incubation, various concentrations of test compounds in the range from 0.1 to 100 μM, were added to the cell cultures, and then the cells were cultured under the same conditions for 24 and 72 h. For each concentration, the experiment was carried out in triplicate.

All compounds were dissolved in DMSO, then diluted with the medium to the required concentration. The final content of DMSO in the well did not exceed 1% and did not have a toxic effect on the cells. The DMSO was also added to the control wells in a volume of 1%. After the incubation time, MTT (3-(4,5-dimethylthiazol-2-yl)-2,5-diphenyltetrazolium bromide, 5 mg/mL) was added to each well and plates were additionally incubated for 2 h (until the characteristic color appeared).

Using a plate analyzer (Cytation3, BioTech Instruments Inc., Winooski, VT, USA), the optical density was determined at 530 nm. The concentration value causing 50% inhibition of cell population growth (IC_50_) was determined from dose-dependent curves.

#### 3.2.4. Determination of Cell Viability

The ability of compounds to suppress anaerobic glycolysis was studied using the Agilent Seahorse XF96e Analyzer (Seahorse Bioscience, North Billerica, MA, USA) by the level of hydrogen proton production in the studied samples on cell lines of neuroblastoma SH-SY5Y using a glycolysis stress test [62]. The rate of extracellular acidification of the medium was measured in real time, which made it possible to assess the intensity of glycolysis in cells by fixing the three main parameters of the glycolytic function: basal glycolysis, glycolytic capacity, and glycolytic reserve.

SH-SY5Y cells in the exponential growth phase were seeded in a 96-well Seahorse cell culture microplate. The planting density of the cell culture was 2 × 10^4^/well. After that, the analyzer was calibrated and, according to the protocol, the sensor cartridge with injection ports was refilled with test reagents to assess the main parameters of glycolysis by modulating cellular metabolism.

The test compounds were added from port A at a concentration of 100 μM. To determine the effect of test compounds on basal glycolysis of neoplastic cells, 10 mM glucose was added from port B. The maximum glycolytic capacity was assessed by injecting oligomycin at a concentration of 1 μM from port C. The difference between maximum glycolytic capacity and basal rate provides information about the glycolytic reserve of cells. As an inhibitor of this process, 25 mM 2-fluoro-2-deoxy-D-glucose was introduced into port D.

#### 3.2.5. Acute Toxicity

All experiments on animals were carried out on the basis of the USU Nursery and Vivarium FRC PCPMC RAS and in accordance with the rules established by the Commission on Bioethics of the FRC PCPMC RAS (protocol No. 5/22 dated May 23, 2022). Studies of acute toxicity were carried out on male hybrid mice BDF_1_ [DBA2 × C57Bl6] weighing from 20 to 24 g. In the experiment, clinically healthy animals were used, which were kept in the same conditions with a 12-h light and free access to water and food. Each experimental group included six animals. To determine acute toxicity, compounds **4e** and **4g** were dissolved in 0.9% NaCl solution and administered intraperitoneally once in the dose range of 5–35 mg/kg. Control animals received 0.9% NaCl solution. Animals were monitored continuously for 4 h on the first day after administration of the test compounds. Then, the death of animals and external signs of intoxication were assessed once a day for 14 days. Calculation of toxic doses was performed using probit analysis.

#### 3.2.6. Antitumor Activity In Vivo

Studies of antitumor activity were carried out on hybrid male mice BDF_1_ [DBA2×C57Bl6] weighing from 22 to 24 g. In the experiment, clinically healthy animals were used, which were kept in the same conditions with a 12-h light and free access to water and food. Each experimental group included six animals. Transplantation of mouse leukemia P388 was performed intraperitoneally with a concentration of 10^6^ cells in 200μLof 0.9% NaCl solution per animal. Compounds **4e** and **4g** were dissolved in 0.9% NaCl solution and administered intraperitoneally once in the dose range 1.25–10 mg/kg and 0.625–5 mg/kg, respectively. The compounds were administered the next day after tumor cell transplantation. Control animals received 0.9% NaCl solution. The effectiveness of therapy was assessed by the increase in the average life span (ILS, %) of the animals.

## 4. Conclusions

A series of novel water-soluble salts based on benzofuroxan derivatives containing aromatic/heterocyclic/aliphatic amines fragments was synthesized through nucleophilic aromatic substitution reaction of 4,6-dichloro-5-nitrobenzofuroxan. Thus, most samples showed good effectiveness of the pre-sowing treatment of seeds for agricultural crops at concentrations of 20–40 mmol. The introduction of an aromatic amine moiety improves the properties so compound **4a** shows activity with less concentration of 10 mmol. An additional advantage of treatment with benzofuroxan derivatives is that in some cases it leads not only to improved seed germination, but also to the suppression of microflora growth.

When studying the anti-cancer activity of the synthesized compounds, it was shown that all substances exhibit a cytotoxic effect in relation to a tumor origincells panel.Compounds containing morpholine fragments, as well as a fragment of N-dimethylpropylamine, **4e** and **4g** showed the highest activity. They also demonstrated the ability to effectively inhibit the glycolysis process in tumor cells. These data allowed us to choosecompounds **4e** and **4g** for further experiments using laboratory animals.

Compound **4e** was found to exhibit significantly less acute toxicity in vivo compared to **4g**. Perhaps **4g** is much more soluble and therefore bioavailable, so it causes more severe intoxication in animals. In addition, test compounds **4e** and **4g** demonstrated in vivo antileukemic activity, as measured by an increase in the mean lifespan of animals with P388 mouse leukemia, compared to an untreated control group.

Thus, water-soluble salts of benzofuroxans can be considered as promising candidates for further development both as anti-cancer and stimulants in seed germination and regulators of microflora crop growth.

## Figures and Tables

**Table 1:** Relative energies (ΔE, kcal/mol), enthalpies (ΔH, kcal/mol), Gibbs energies (ΔG, kcal/mol) and HOMO-LUMO gaps (eV) of the products **3d** and **5d** with HCl compared to the initial reagents 4,6-dichloro-5-nitrobenzofuroxan **1** with 2-morpholinoethanamine **2d** (Scheme 2).

Reaction Products	ΔE	ΔH	ΔG	HOMO-LUMO
**3d**+HCl	22.51	22.08	18.73	-
**5d**+HCl	23.93	23.47	20.49	-
**3d**	0.00	0.00	0.00	3.07
**5d**	−1.42	−1.39	−1.76	3.04

**Table 2:** Relative energies (ΔE, kcal/mol), enthalpies (ΔH, kcal/mol), Gibbs free energies (ΔG, kcal/mol) and HOMO-LUMO gaps (eV) for reagents (**1+2d**), pre-reaction complexes **[1+2d],** transition states **TS,** intermediates **IM** and products **3d** and **5d** of the reaction of 4,6-dichloro-5-nitrobenzofuroxan **1** with 2-morpholinoethanamine **2d**.

Reaction Products	ΔE	ΔH	ΔG	HOMO-LUMO
**1+2d**	0.00	0.00	0.00	-
**1 way**				
**[1+2d]a**	−5.61	−4.85	1.81	-
**TS1a**	10.96	10.38	24.32	3.12
**IM1**	3.24	2.67	17.20	3.16
**TS1**	5.28	4.64	19.42	2.48
**2 way**				
**[1+2d]b**	−5.86	−5.16	2.63	-
**TS2**	22.31	21.75	36.16	3.04
**IM2a**	−23.88	−23.38	−13.44	3.37
**TS2b**	11.84	11.42	24.99	3.05
**IM2b+HCl**	−18.17	−17.77	−15.16	3.07
**TS2a+HCl**	−1.40	−1.27	2.71	3.19

**Table 3:** Effect of seed treatment with test compounds on sowing qualities.

Agricultural Crop	Line Number	Estimated Parameters		Control	4a, Concentration, mmol		4d, Concentration, mmol			4e, Concentration, mmol			4g, Concentration, mmol		
					10	50	10	20	40	10	20	40	10	20	40
wheat	1.	germination energy, %		82	97	95	47.6	71.4	98	33.3	97	98	42.8	80.9	80.9
	2.	laboratory germination, %		85.7	96.5	96.5	47.6	71.4	98.5	42.8	97.5	99	47.6	90.4	80.9
	3.	the growth rate of seedlings, %		++	++	++	++	++	+++	++	+++	+++	++	+++	++
	4.	growth rate of microorganisms	mushrooms	+++	+++	+++	+++	+++	++	+++	+	+	+++	+++	+++
	5.		bacteria	++			+	+	+	++	++	+	+	++	++
alfalfa	6.	germination energy, %		76	83	78	43.3	63.3	78	46.6	78.5	73	56.6	43.3	80
	7.	laboratory germination, %		76	84.5	79.5	50	73.3	80.5	46.6	79.5	74	60	63.3	80
	8.	the growth rate of seedlings, %		++	+++	+++	+	++	+++	++	+++	+++	++	+++	++
	9.	growth rate of microorganisms	mushrooms	+	+	+	-	-	+++	+++	+++	+++	+	+++	++
	10.		bacteria				+++	+++	+	+	+	+	+++	++	++
barley	11.	germination energy, %		95.2	96	95	71.4	66.6	95	85.7	96	94	52.3	100	100
	12.	laboratory germination, %		95.2	99	97.5	71.4	66.6	98.5	85.7	98	99	52.3	100	100
	13.	the growth rate of seedlings, %		+++	++	++	++	+++	+++	+++	+++	+++	++	+++	+++
	14.	growth rate of microorganisms	mushrooms	++	++	++	+++	+++	+++	+++	+++	+++	+++	+++	+++
	15.		bacteria	++			+	+++	+	+	+	+	++	++	++
rice	16.	germination energy, %		71.4	96	99	52.3	47.6	99	38	98.5	99	38	71.4	76.1
	17.	laboratory germination, %		95.2	96.5	99	52.3	95.2	99	38	99	99	47.6	100	95.2
	18.	the growth rate of seedlings, %		++	++	++	++	++	+++	++	+++	+++	++	++	+++
	19.	growth rate of microorganisms	mushrooms	+++	+++	+++	+++	+++	+++	+++	+++	+++	+++	+++	++
	20.		bacteria	+++			+	+	++	+	+	++	+++	+++	++
sorghum	21.	germination energy, %		52.3	47.5	52.5	33.3	76.1	58.5	33.3	50.5	50.5	36.6	61.9	57.2
	22.	laboratory germination, %		57	49.5	56.5	40	80.9	61.5	63.3	56	52.5	50	71.4	80.9
	23.	the growth rate of seedlings, %		++	+++	+++	++	++	+++	+	+++	+++	++	++	++
	24.	growth rate of microorganisms	mushrooms	++	++	++	++	++	+++	++	+++	+++	++	++	++
	25.		bacteria	+++			++	+++	++	+++	++	++	++	+++	+++

**Table 4:** Cytotoxic potential of water-soluble salts based on benzofuroxan.

Incubation–24 h					
	**SH-SY5Y**	**Hep2**	**A549**	**HeLa**	**Hek 293**
**4a**	43.96 ± 1.09	98.53 ± 2.54	95.54 ± 1.01	40.17 ± 2.73	38.62 ± 0.10
**4b**	45.18 ± 2.43	97.04 ± 2.08	99.21 ± 3.38	46.09 ± 0.56	35.01 ± 1.35
**4d**	24.28 ± 1.91	91.28 ± 1.40	>100	40.53 ± 1.90	40.84 ± 2.84
**4e**	24.07 ± 1.22	83.00 ± 1.37	90.25 ± 3.43	>100	99.01 ± 2.09
**4g**	20.89 ± 0.82	70.28 ± 1.28	82.01 ± 1.54	97.81 ± 3.31	21.95 ± 0.39
**Incubation—72 h**					
	**SH-SY5Y**	**Hep2**	**A549**	**HeLa**	**Hek 293**
**4a**	35.12 ± 3.85	90.14 ± 3.10	92.10 ± 3.58	32.90 ± 0.56	16.13 ± 1.86
**4b**	32.08 ± 1.41	93.06 ± 2.30	90.05 ± 2.04	38.12 ± 1.21	27.19 ± 2.50
**4d**	22.79 ± 1.90	89.94 ± 3.12	98.21 ± 0.10	35.47 ± 0.12	28.10 ± 0.93
**4e**	20.21 ± 0.72	70.21 ± 0.54	77.14 ± 1.36	93.8 ± 2.53	86.49 ± 5.75
**4g**	17.07 ± 0.03	58.03 ± 2.90	64.19 ± 2.12	83.1 ± 4.29	12.03 ± 1.12

**Table 5:** Acute toxicity parameters of compounds **4e** and **4g** after intraperitoneal administration.

Compound	MTD	LD_16_	LD_50_	LD_84_	LD_100_
**4e**	10	18.2	22.00 ± 1.33	24.6	30
**4g**	5	9.4	13.75 ± 1.73	20.3	25

**Table 6:** The body weight changes of BDF_1_ hybrid mice after a single intraperitoneal injection of **4e**.

Dose, mg/kg	Body Weight, g					
	0 Day	1 Day	2 Days	3 Days	7 Days	14 Days
10	20.25 ± 0.39	20.39 ± 0.47	20.51 ± 0.40	20.88 ± 0.31	21.58 ± 0.51	22.12 ± 0.62
15	21.48 ± 0.47	21.45 ± 0.51	21.56 ± 0.49	21.62 ± 0.59	22.02 ± 0.47	22.66 ± 0.38
20	21.45 ± 0.52	21.44 ± 0.71	21.88 ± 0.81	22.02 ± 0.74	22.74 ± 0.58	23.01 ± 0.65
25	22.27 ± 0.29	21.98	21.81	21.89	22.15	22.74
30	21.92 ± 0.13	-	-	-	-	-
control	21.55 ± 0.28	21.43 ± 0.56	21.67 ± 0.77	21.99 ± 0.79	22.80 ± 0.44	23.96 ± 0.68

**Table 7:** Body weight changes of BDF_1_ hybrid mice after a single intraperitoneal injection of **4g**.

Dose, mg/kg	Body Weight, g					
	0 Day	1 Day	2 Days	3 Days	7 Days	14 Days
5	21.88 ± 0.45	21.59 ± 0.55	21.64 ± 0.71	21.72 ± 0.79	22.12 ± 0.75	23.19 ± 1.21
10	21.99 ± 0.21	21.54 ± 0.41	21.60 ± 0.52	21.86 ± 0.66	22.84 ± 1.06	23.70 ± 1.12
15	21.86 ± 0.57	21.36 ± 2.51	21.60 ± 2.43	22.11 ± 3.03	22.41 ± 2.74	23.15 ± 2.14
20	22.37 ± 0.80	21.99 ± 3.09	21.77 ± 3.79	21.85 ± 3.18	22.81 ± 3.89	23.41 ± 3.01
25	21.87 ± 0.74	-	-	-	-	-
control	21.55 ± 0.28	21.43 ± 0.56	21.67 ± 0.77	21.99 ± 0.79	22.80 ± 0.44	23.96 ± 0.68

## Supplementary Materials

The following supporting information can be downloaded at: https://www.mdpi.com/article/10.3390/ijms232314902/s1.
