# Supplementary material for: Water-Soluble Salts Based on Benzofuroxan Derivatives—Synthesis and Biological Activity
Source: Int J Mol Sci. 2022 Nov 28;23(23):14902. doi: 10.3390/ijms232314902 (PMC9739695; doi:10.3390/ijms232314902)
Supplement: Supplementary file 1 [file ijms-23-14902-s001.zip › ijms-2026580-supplementary.pdf]

Supplementary Materials

Water-Soluble Salts Based on Benzofuroxan Derivatives. Synthesis and Properties

Elena Chugunova [1,*], Victoria Matveeva [1], Alena Tulesinova [2], Emil Iskanderov [3], Nurgali Akylbekov [4], Alexey Dobrynin [1], Ayrat Khamatgalimov [1], Alexander Burilov [1], Lyazat Boltayeva [6], Bakhytzhan Duisembekov [6], Mukhtar Zhanakov [7], Yulia Aleksandrova[1,8], Margarita Neganova[1,8], Nurbol Appazov [4,5], Tatyana Sashenkova[9], Elena Klimanova[9], Ugulzhan Allayarova[9], Anastasia Balakina[9] and Denis Mishchenko[9,10,11]

1 Arbuzov Institute of Organic and Physical Chemistry, FRC Kazan Scientific Center, Russian Academy of Sciences, Akad. Arbuzov street 8, Kazan 420088, Russia
2 The Kazan National Research Technological University, Karl Marx street, 68, Kazan 420015, Russia
3 Kazan Federal University, Kremlyovskaya street 18, Kazan 420008, Russia
4 Korkyt Ata Kyzylorda University, Laboratory of Engineering Profile "Physical and Chemical Methods of Analysis", Aitekebie street 29A, Kyzylorda 120014, Kazakhstan
5 I. Zhakhaev Kazakh Scientific Research Institute of Rice Growing, Abay Avenue 25B, Kyzylorda 120008, Kazakhstan
6 Kazakh Scientific Research Institute of Plant Protection and Quarantine named after Zhazken Zhiembayev LLP, Kultobe street 1, Almaty A30M0H6, Kazakhstan
7 L.N. Gumilyov Eurasian National University, Satpayev street 2, Astana 010008, Kazakhstan
8 Institute of Physiologically Active Compounds at Federal Research Center of Problems of Chemical Physics and Medicinal Chemistry, Russian Academy of Sciences, Severny proezd 1, Chernogolovka 142432, Russia
9 Federal Research Center of Problems of Chemical Physics and Medicinal Chemistry RAS, Academician Semenov avenue 1, Chernogolovka 142432, Russia
10 M.V. Lomonosov MSU, Faculty of Fundamental Physical-Chemical Engineering, Leninskie gory 1, Moscow 119991, Russia
11 Moscow Regional State University in Chernogolovka, Biomedical Institute of the Scientific and Educational Center, Vera Voloshina street, 24, Mytishchi 141014,Russia
* Correspondence: chugunova.e.a@gmail.com (E.C.); nurgali_089@mail.ru (N.A.); Tel.: +7-843-272-7324 (E.C.); +7-724-223-1041 (N.A.)

Contents

Copies of NMR spectra 2-24
X-Ray Crystallography Data 25-26

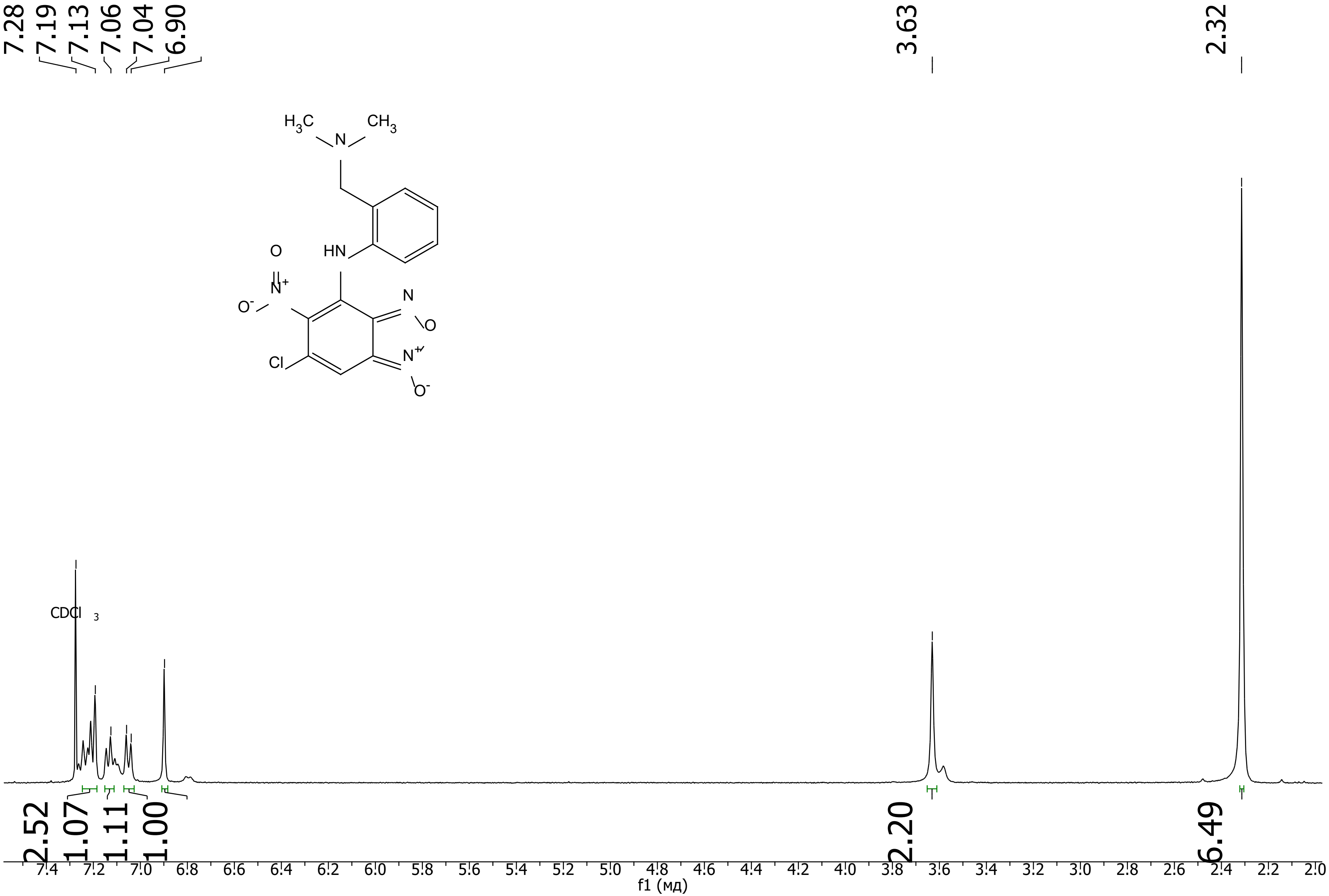

Figure S1. ^{1}H NMR ($CDCl_3$, 400 MHz, 303 K of compound **3a**.

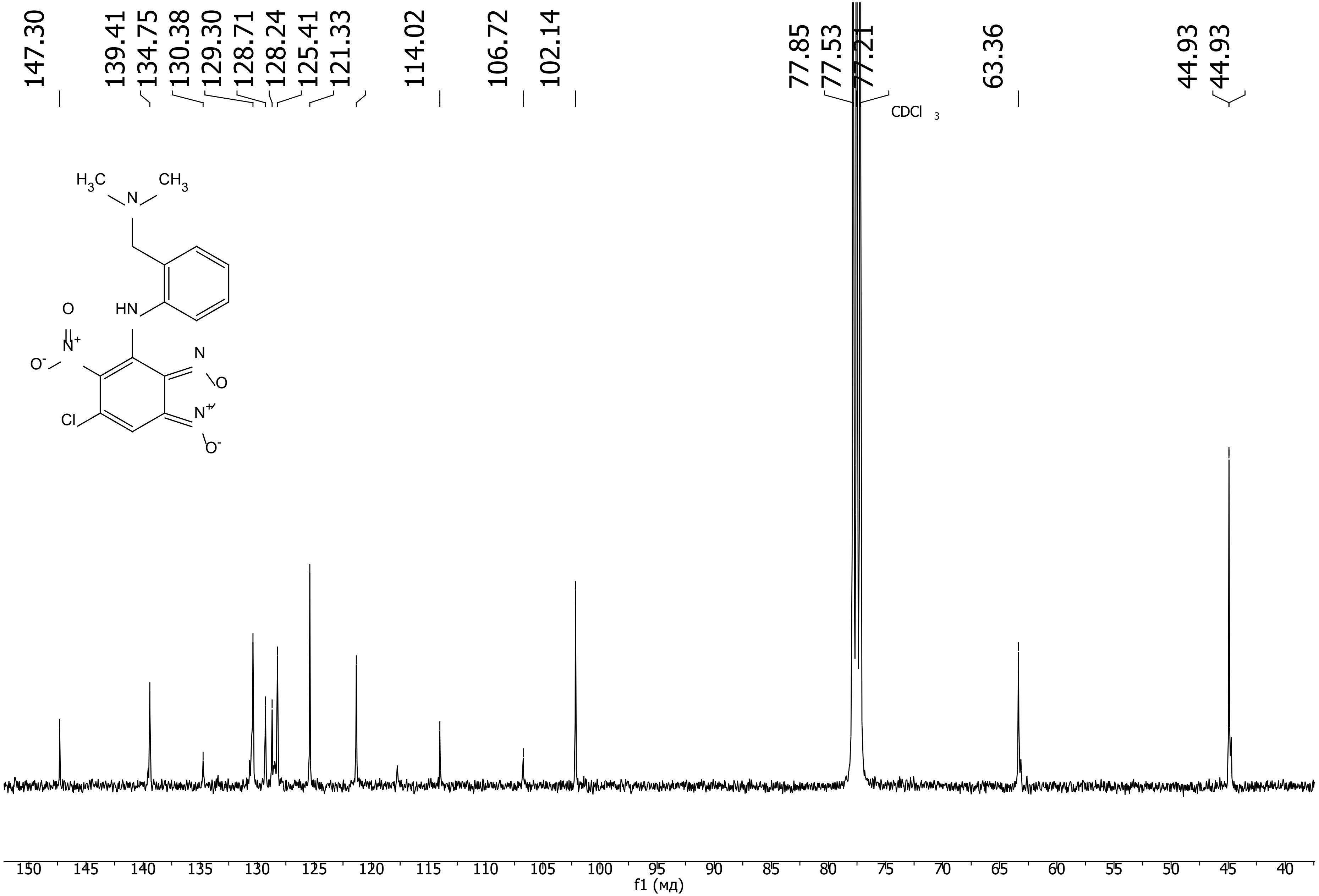

Figure S2. ^{13}C NMR ($CDCl_3$, 101 MHz, 303 K) of compound **3a**.

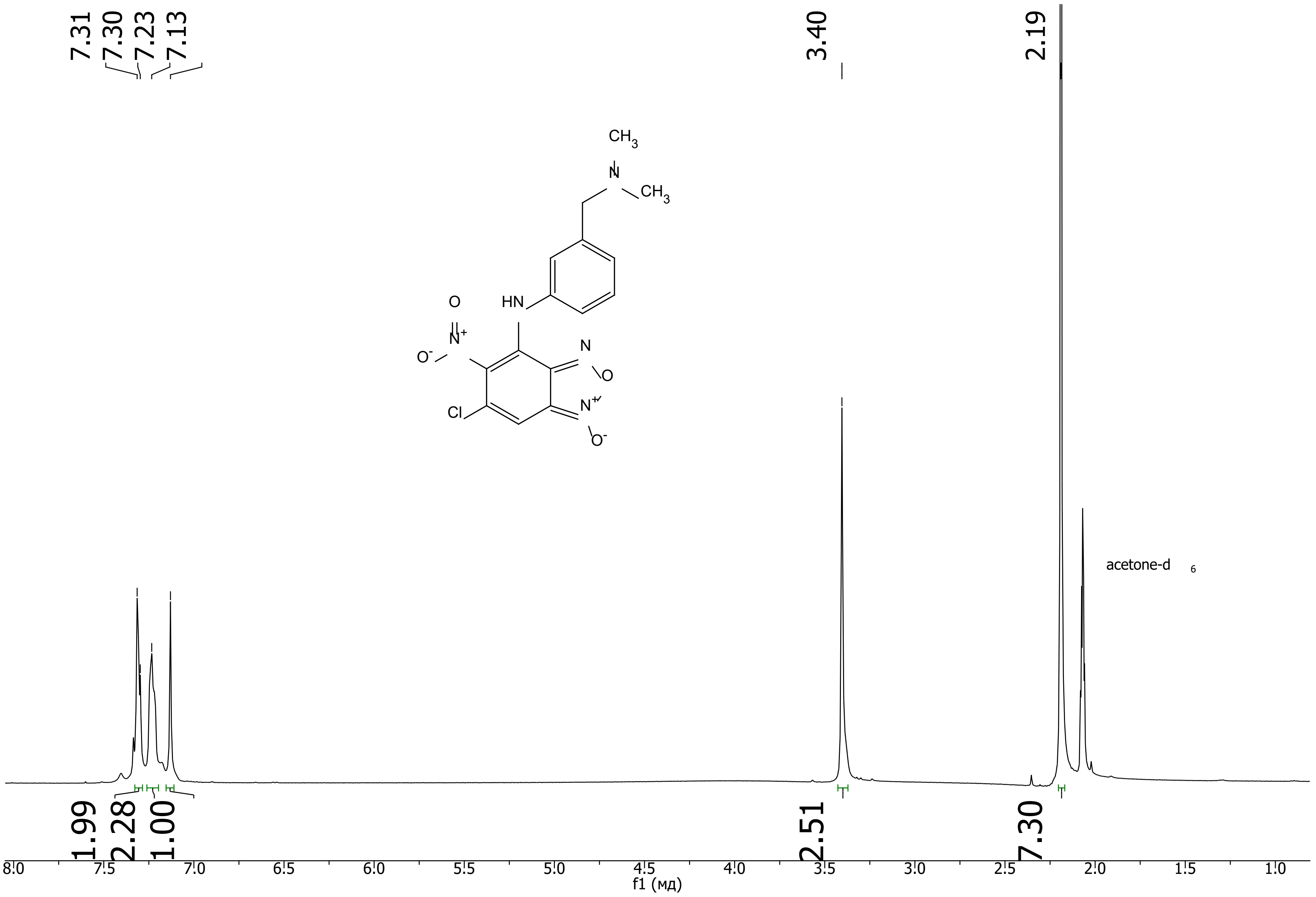

Figure S3. ^{1}H NMR (acetone-d$_6$, 400 MHz, 303 K) of compound **3b**.

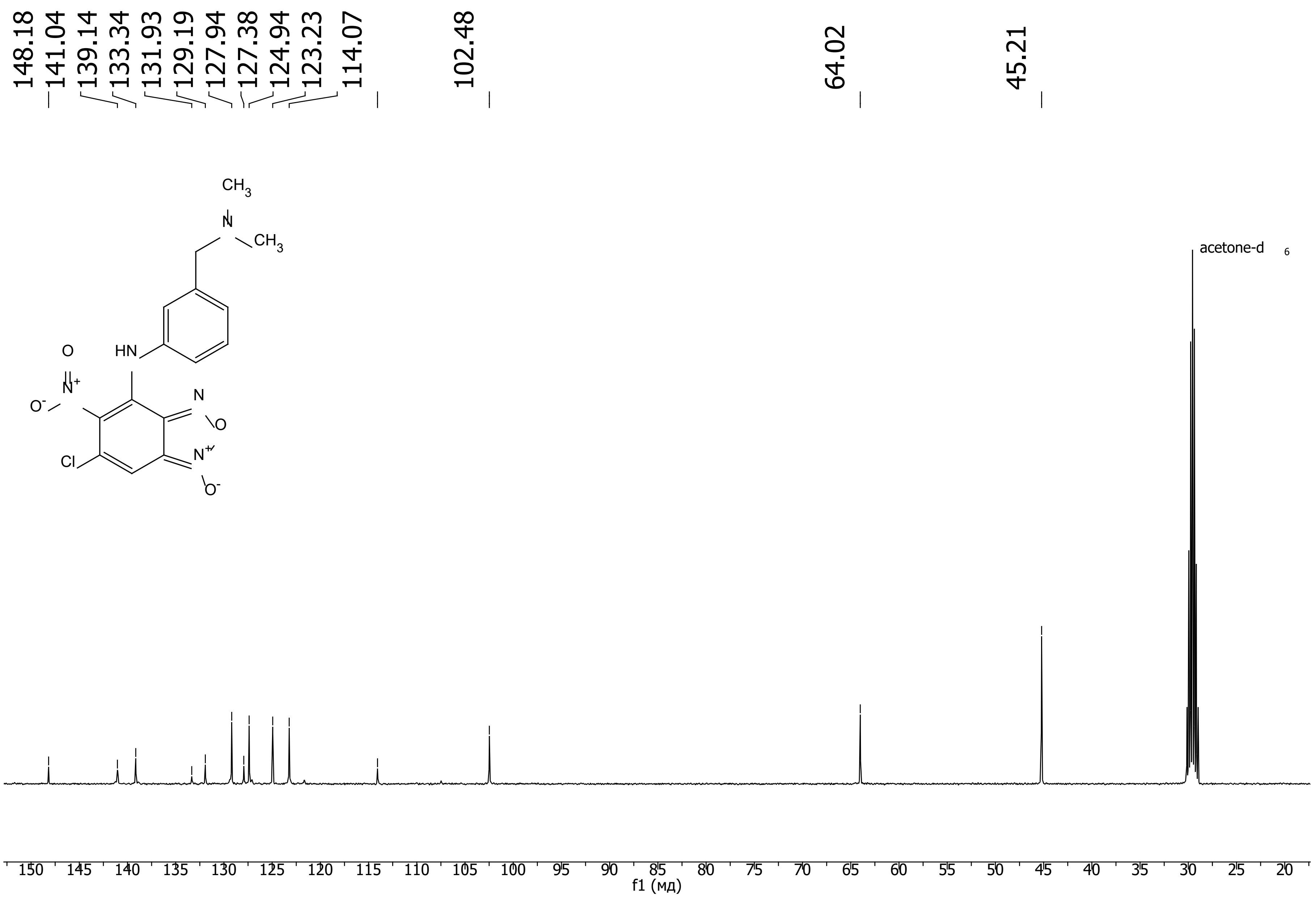

Figure S4. ^{13}C NMR (acetone-d_6, 101 MHz, 303 K) of compound **3b**.

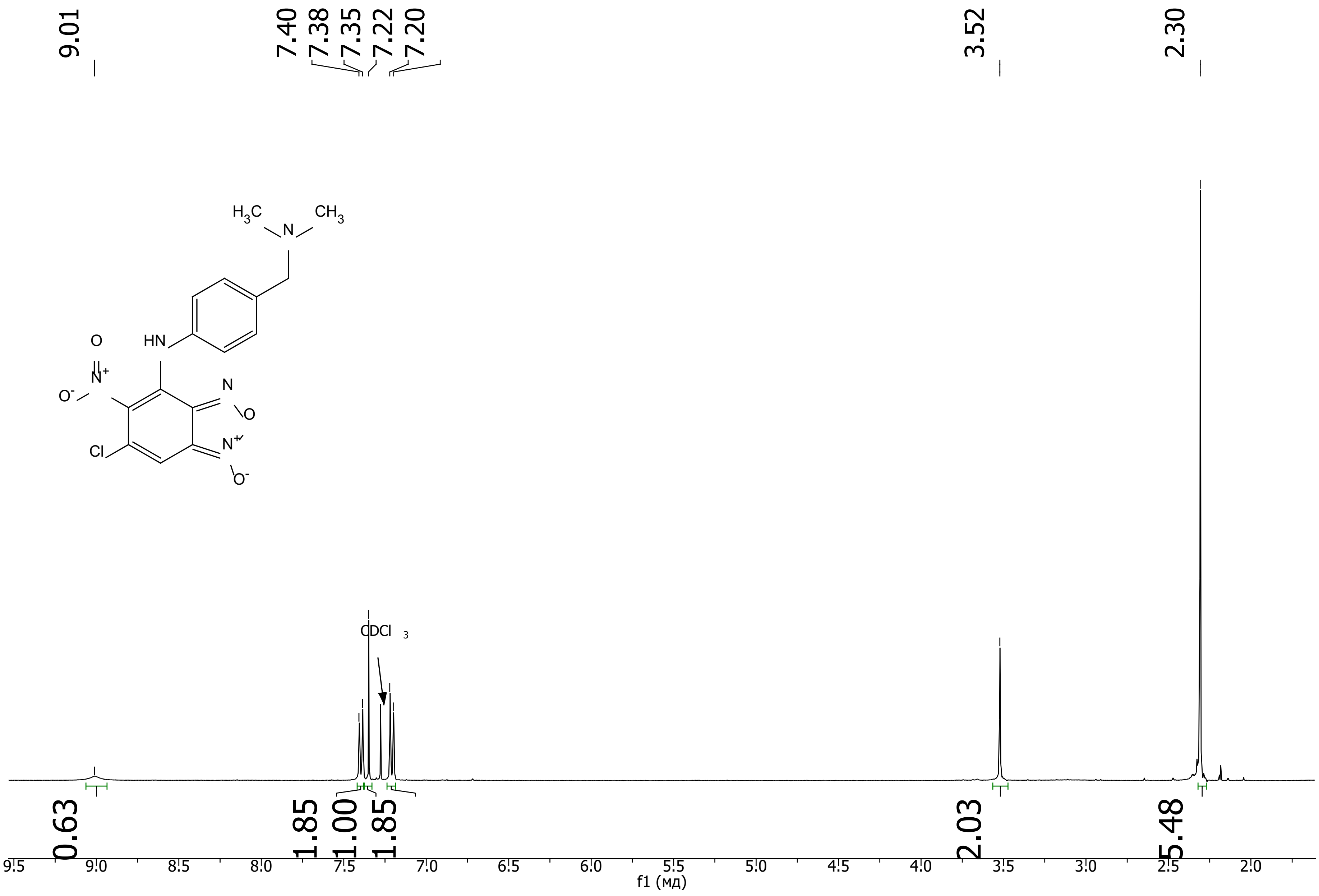

Figure S5. ^{1}H NMR ($CDCl_3$, 400 MHz, 303 K) of compound **3c**.

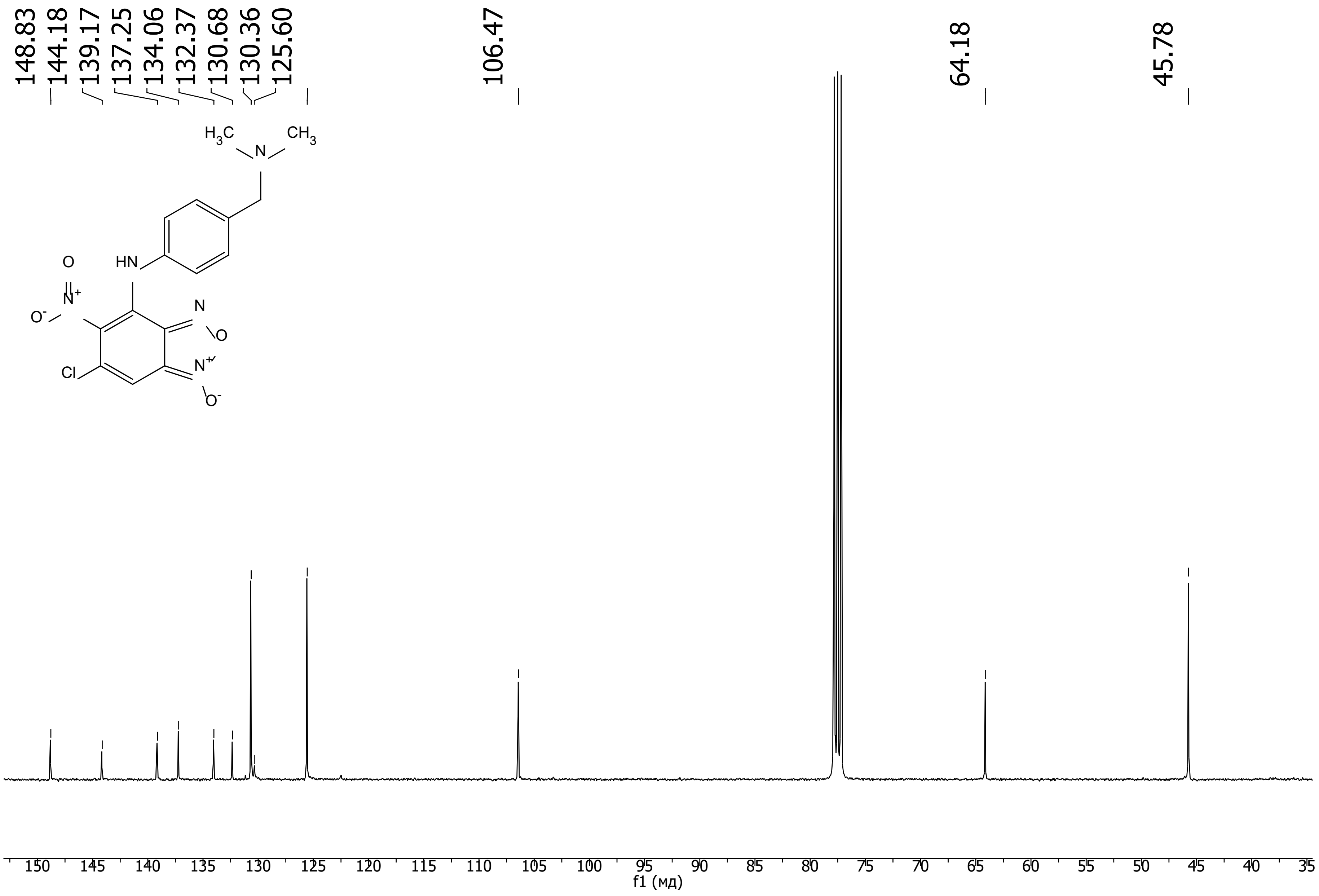

Figure S6. ^{13}C NMR ($CDCl_3$, 101 MHz, 303 K) of compound **3c**.

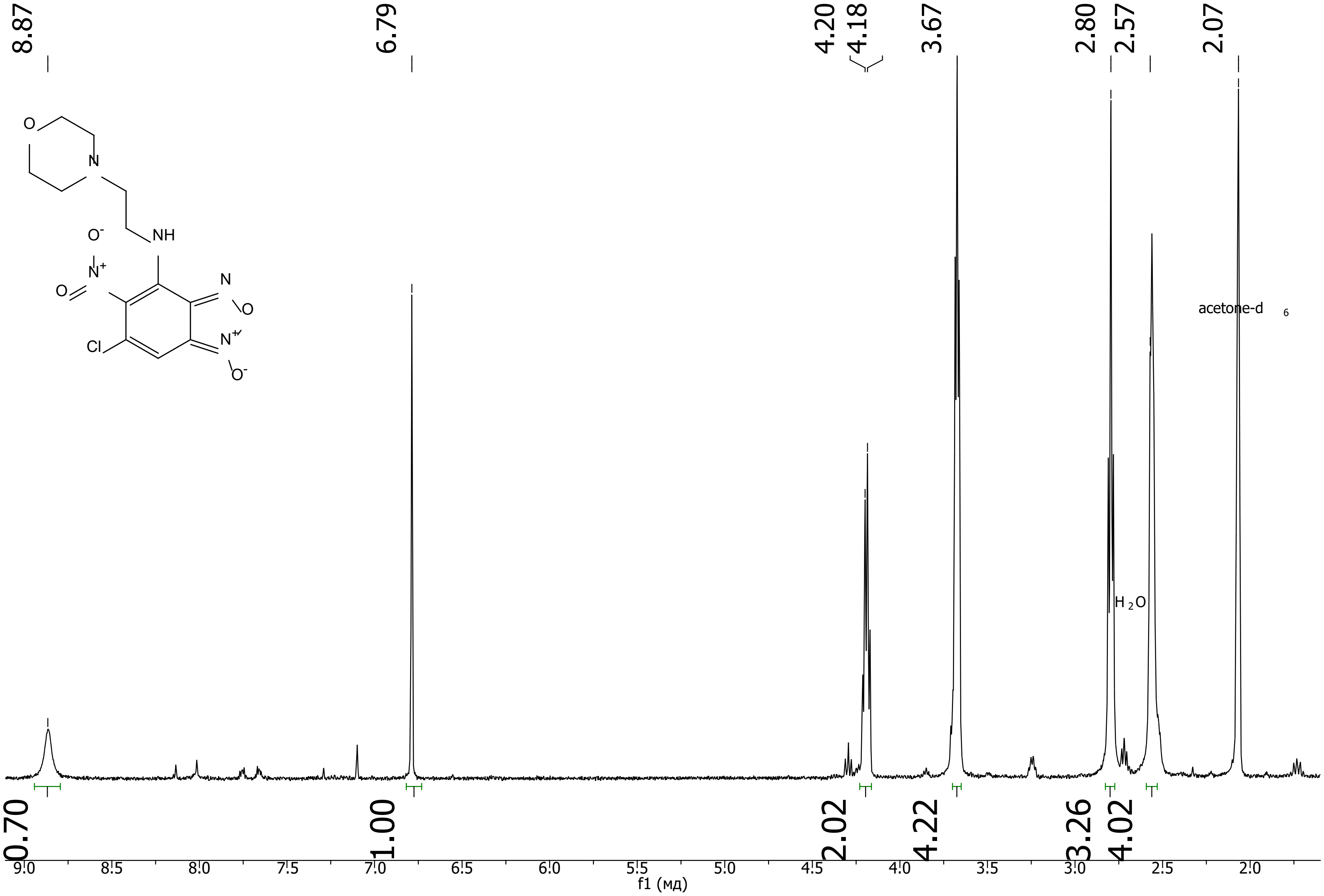

Figure S7. ^{1}H NMR (acetone-d_6, 400 MHz, 303 K) of compound **3d**.

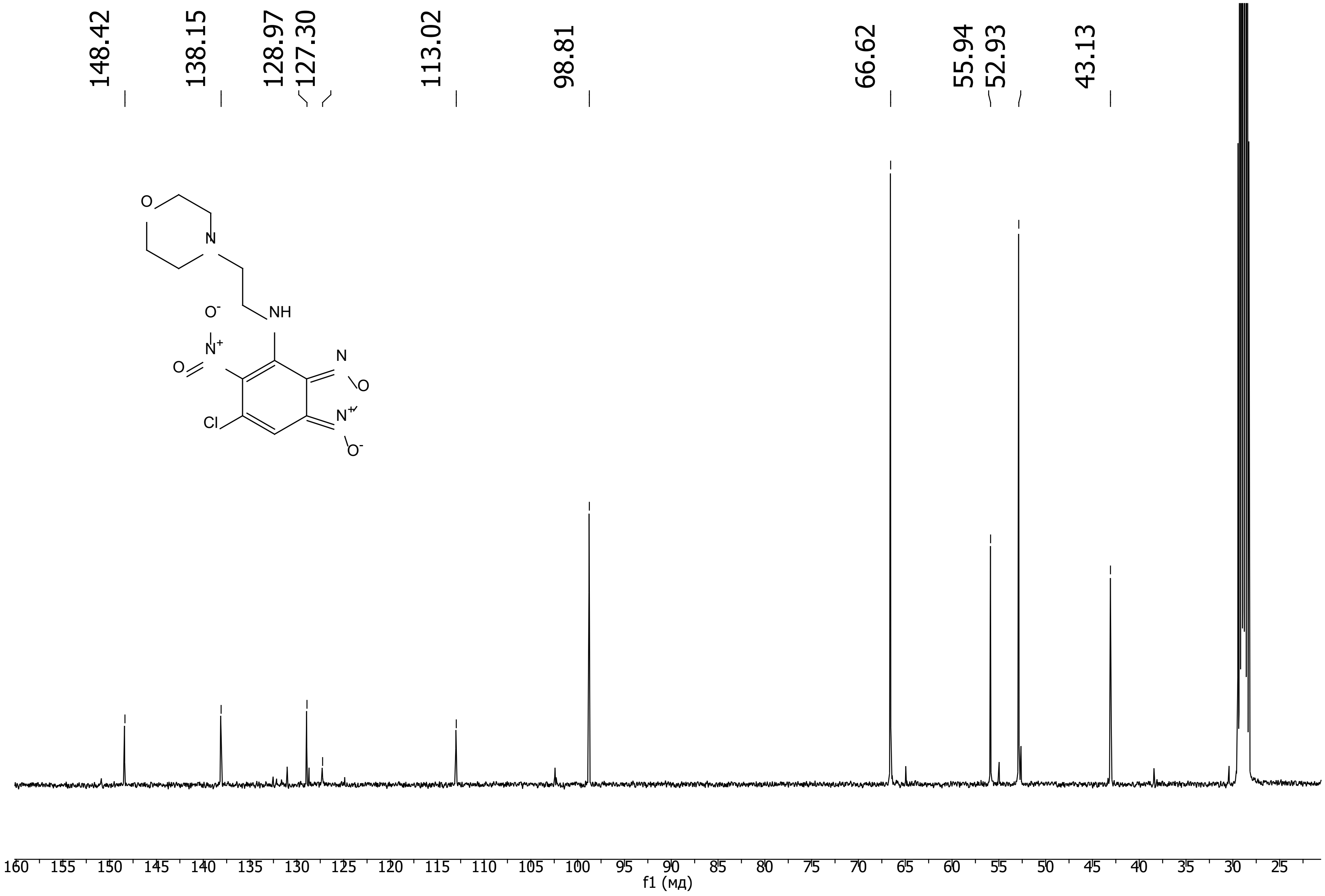

Figure S8. $^{13}C\{1H\}$ NMR (acetone-d_6, 101 MHz, 303 K) of compound **3d**.

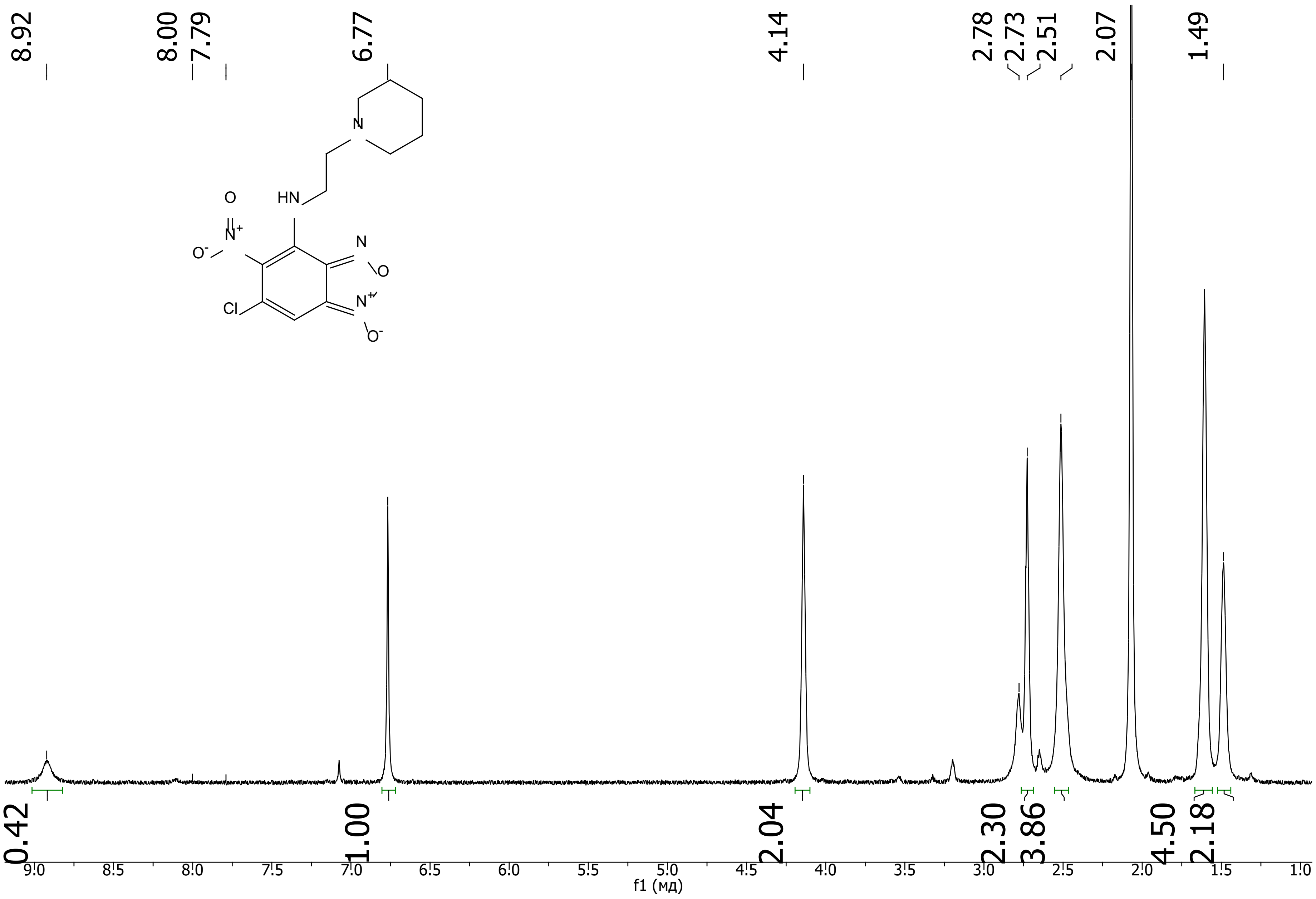

Figure S9. ^{1}H NMR (acetone-d_6, 600 MHz, 303 K) of compound **3f**.

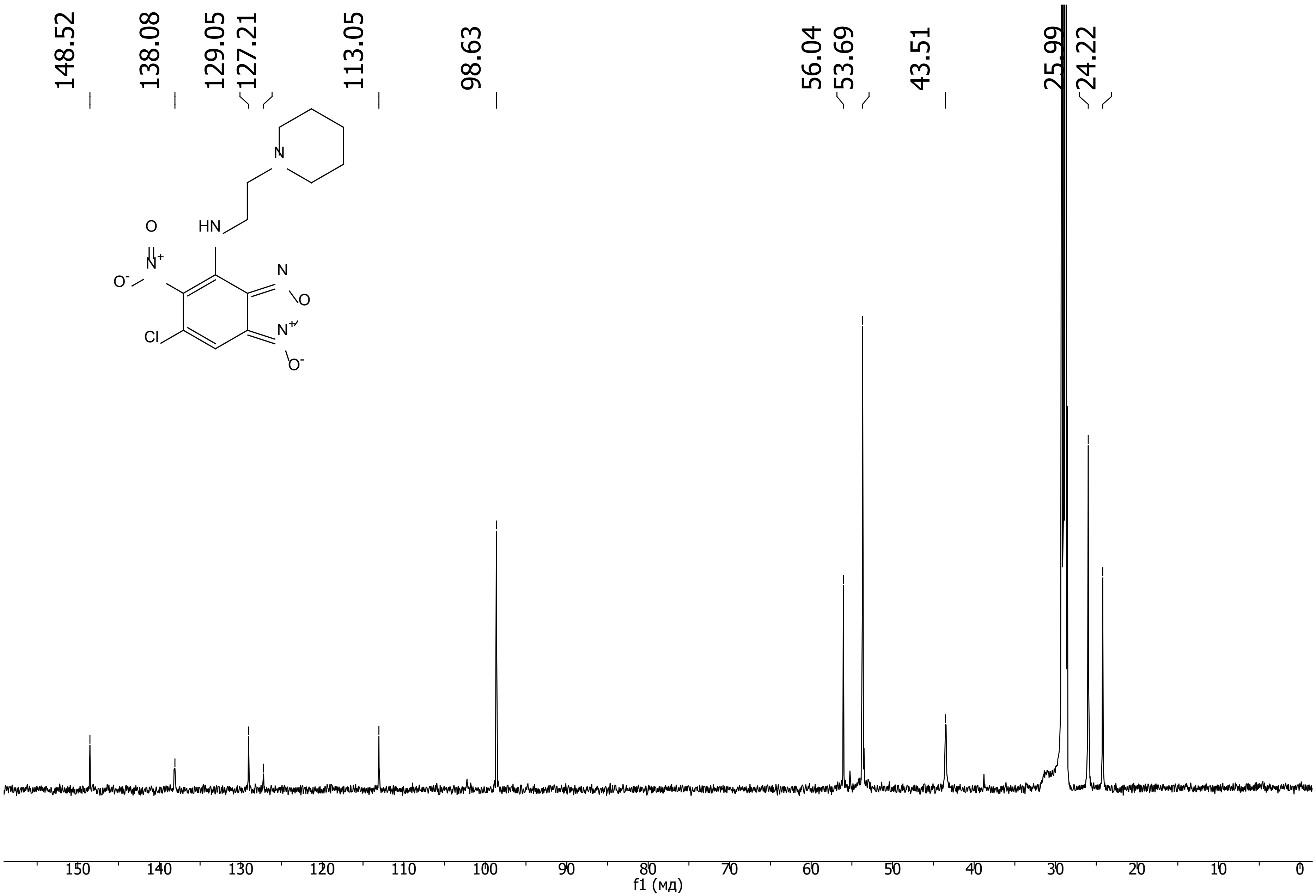

Figure S10. ^{13}C NMR (acetone-d_6, 151 MHz, 303 K) of compound **3f**.

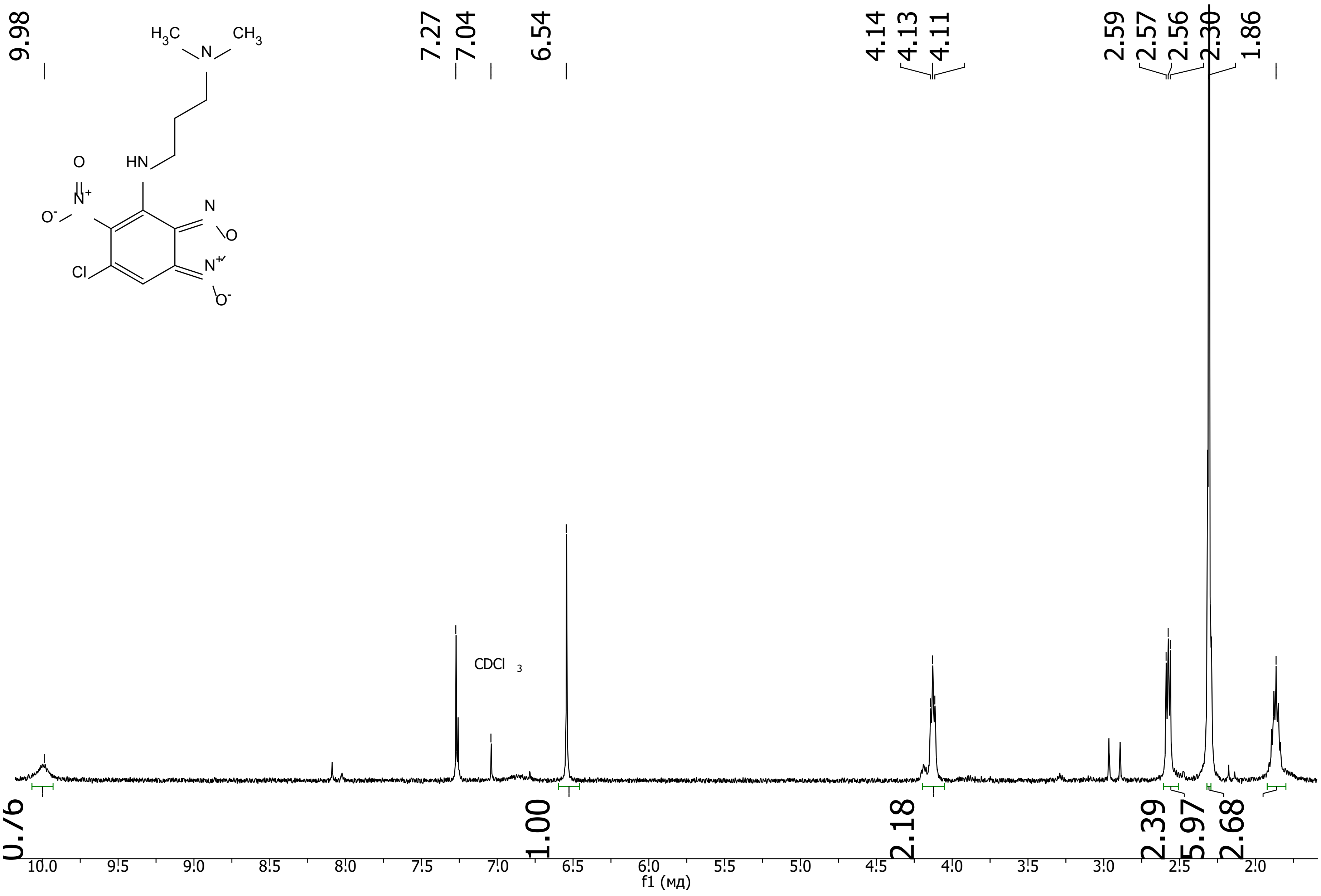

Figure S11. ^{1}H NMR ($CDCl_3$, 400 MHz, 303 K) of compound **3g**.

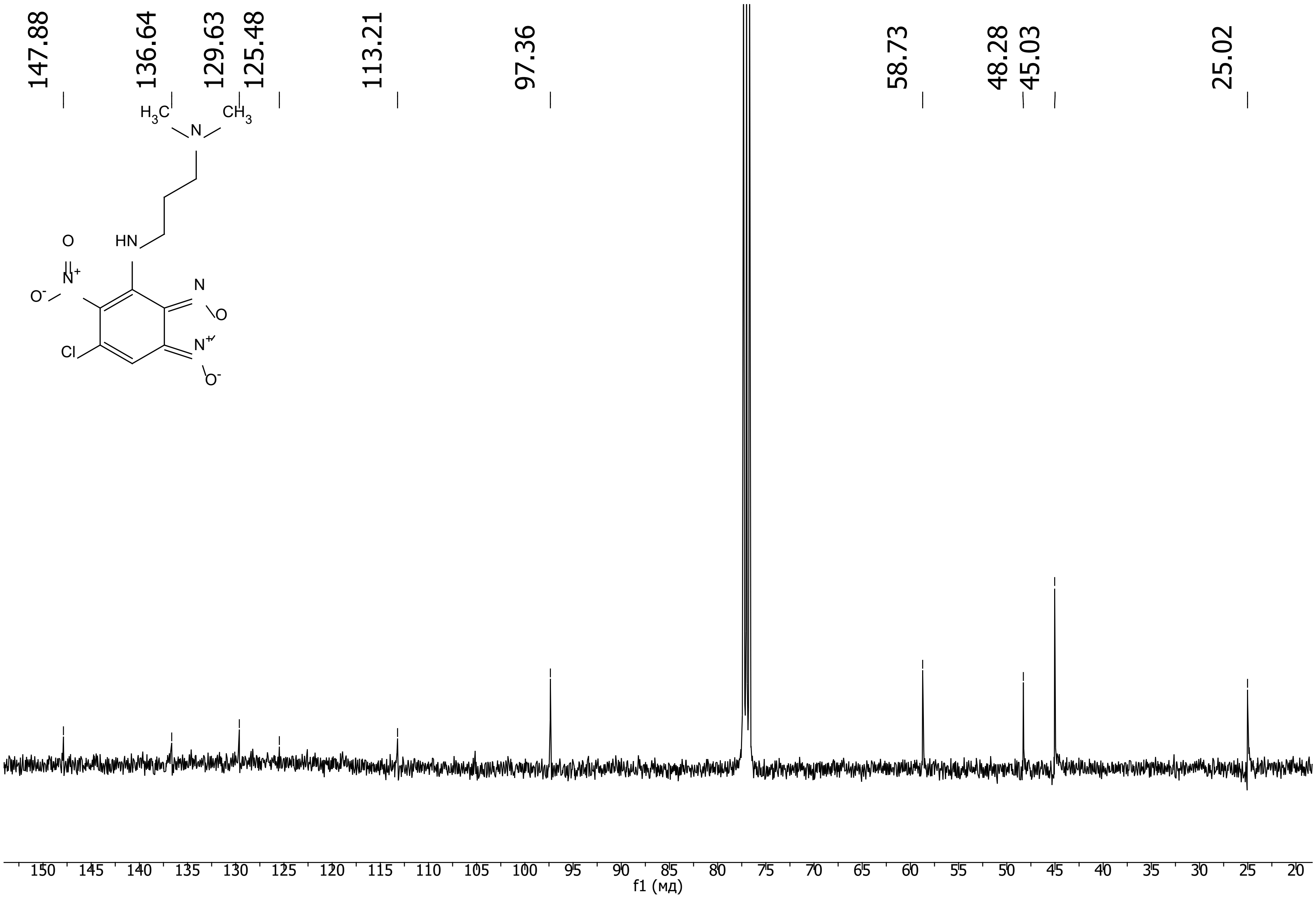

Figure S12. ^{13}C NMR (acetone-d_6, 101 MHz, 303 K) of compound **3g**.

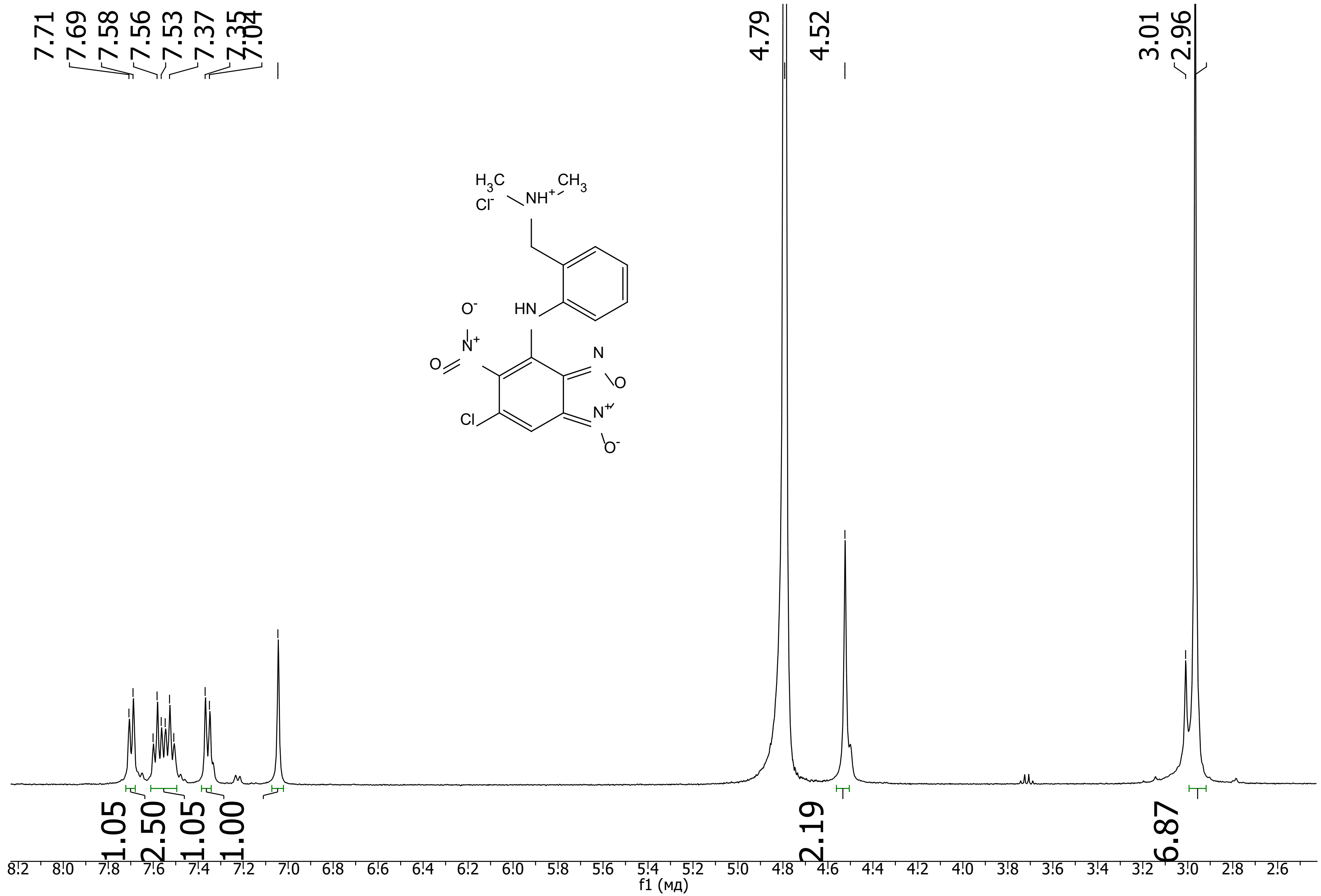

Figure S13. ^{1}H NMR (D_2O, 600 MHz, 303 K) of compound **4a**.

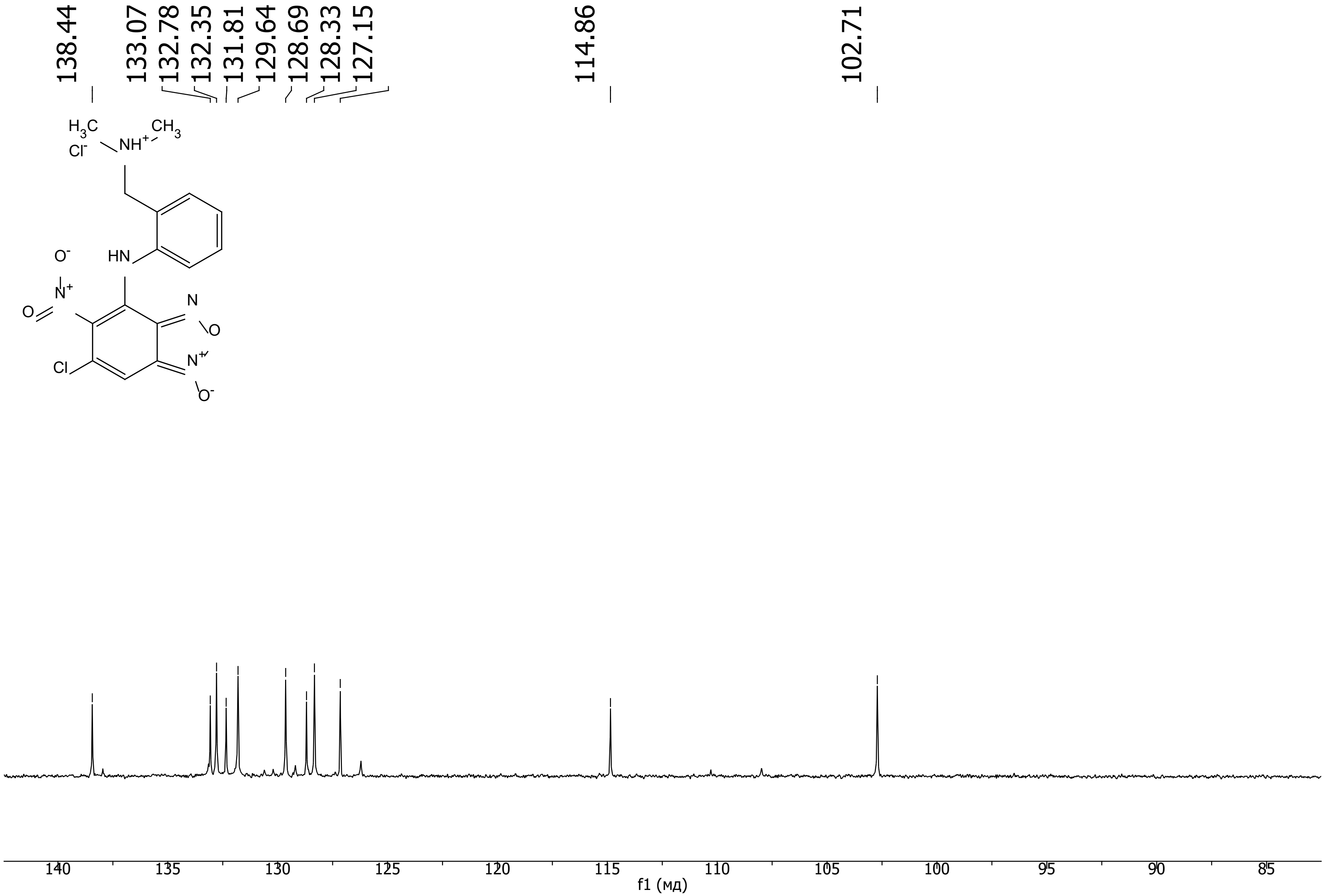

Figure S14. ^{13}C NMR (D_2O, 151 MHz, 303 K) of compound **4a**.

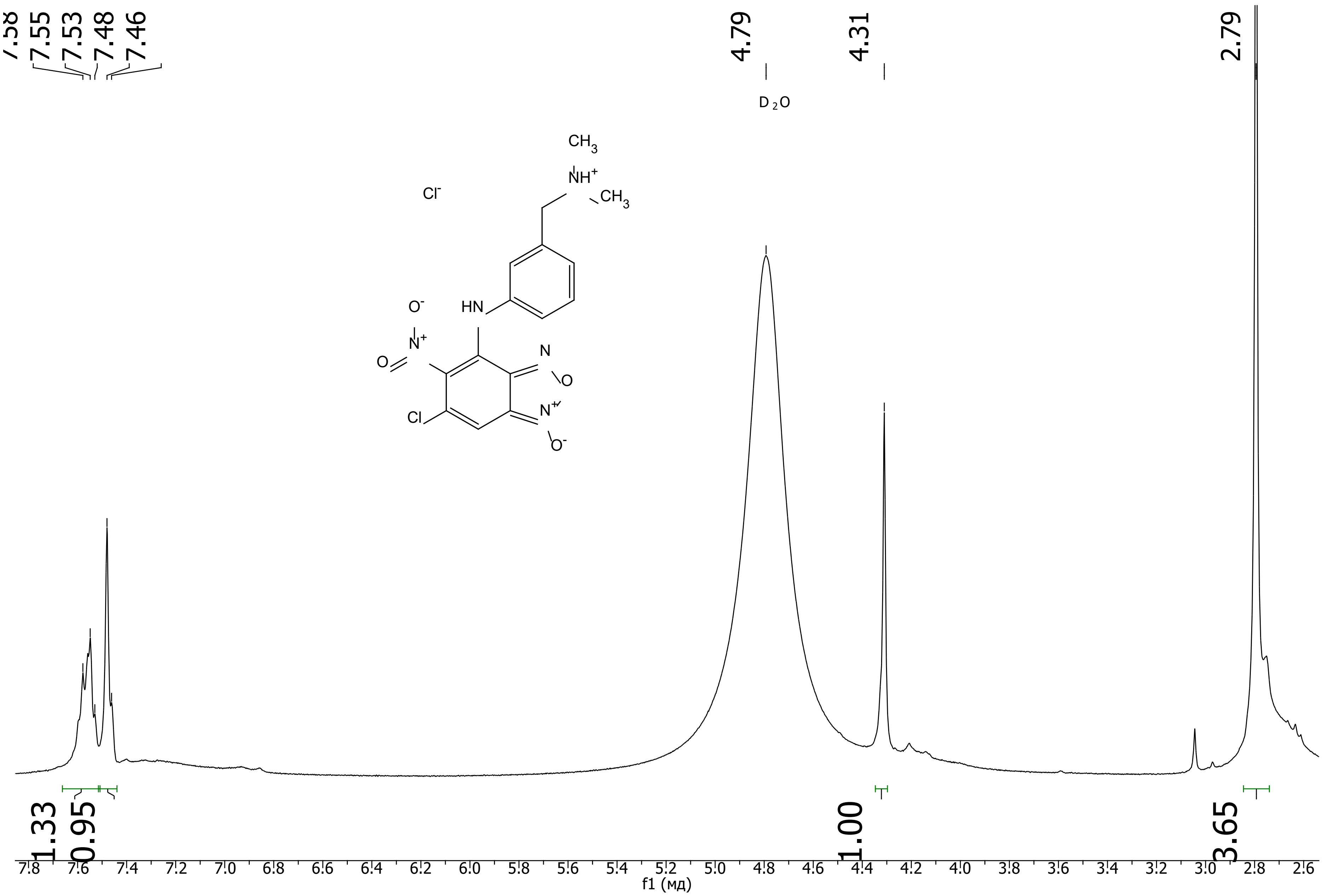

Figure S15. ^{1}H NMR (D_2O, 600 MHz, 303 K) of compound **4b**.

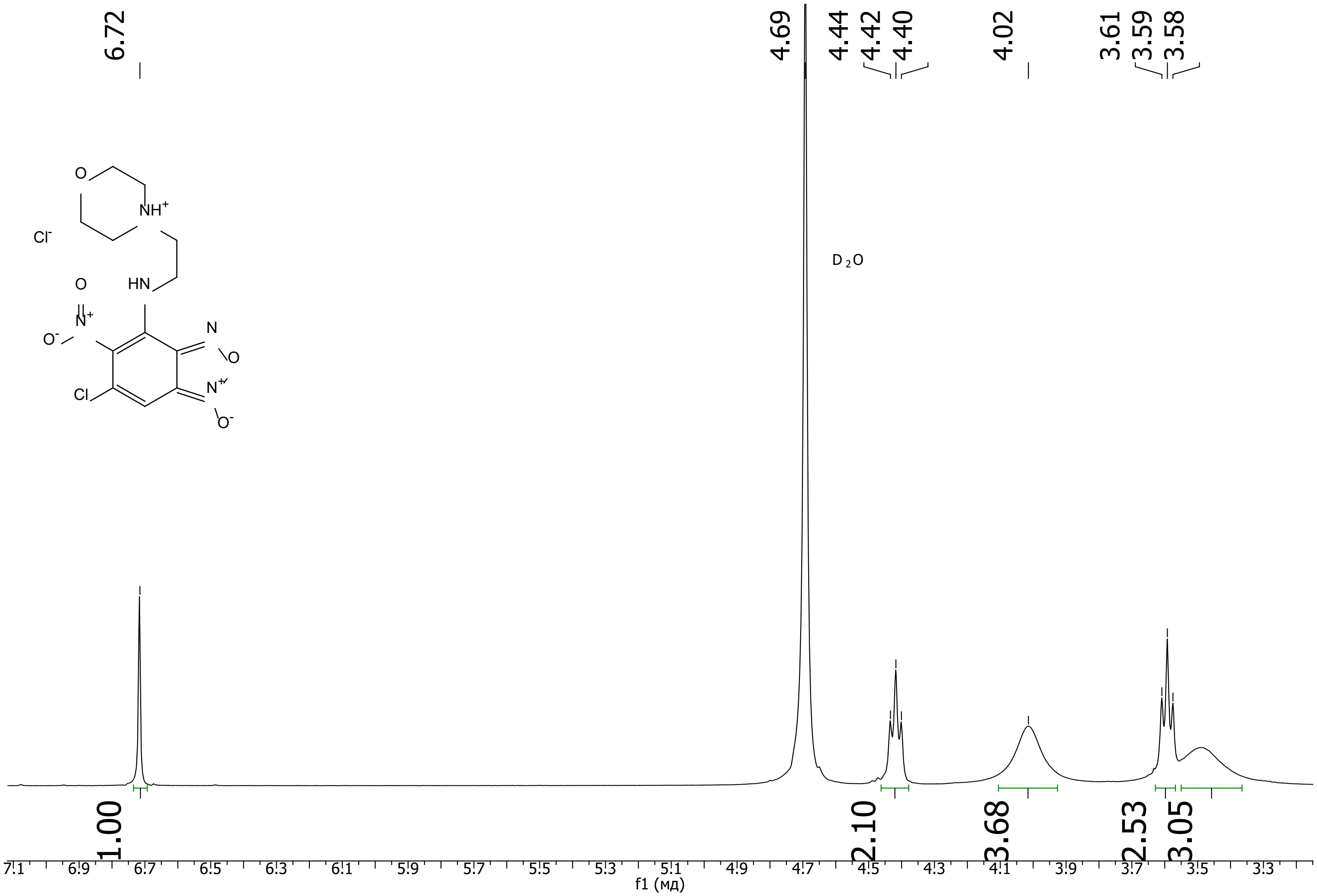

Figure S16. ^{1}H NMR (D_2O, 400 MHz, 303 K) of compound **4d**.

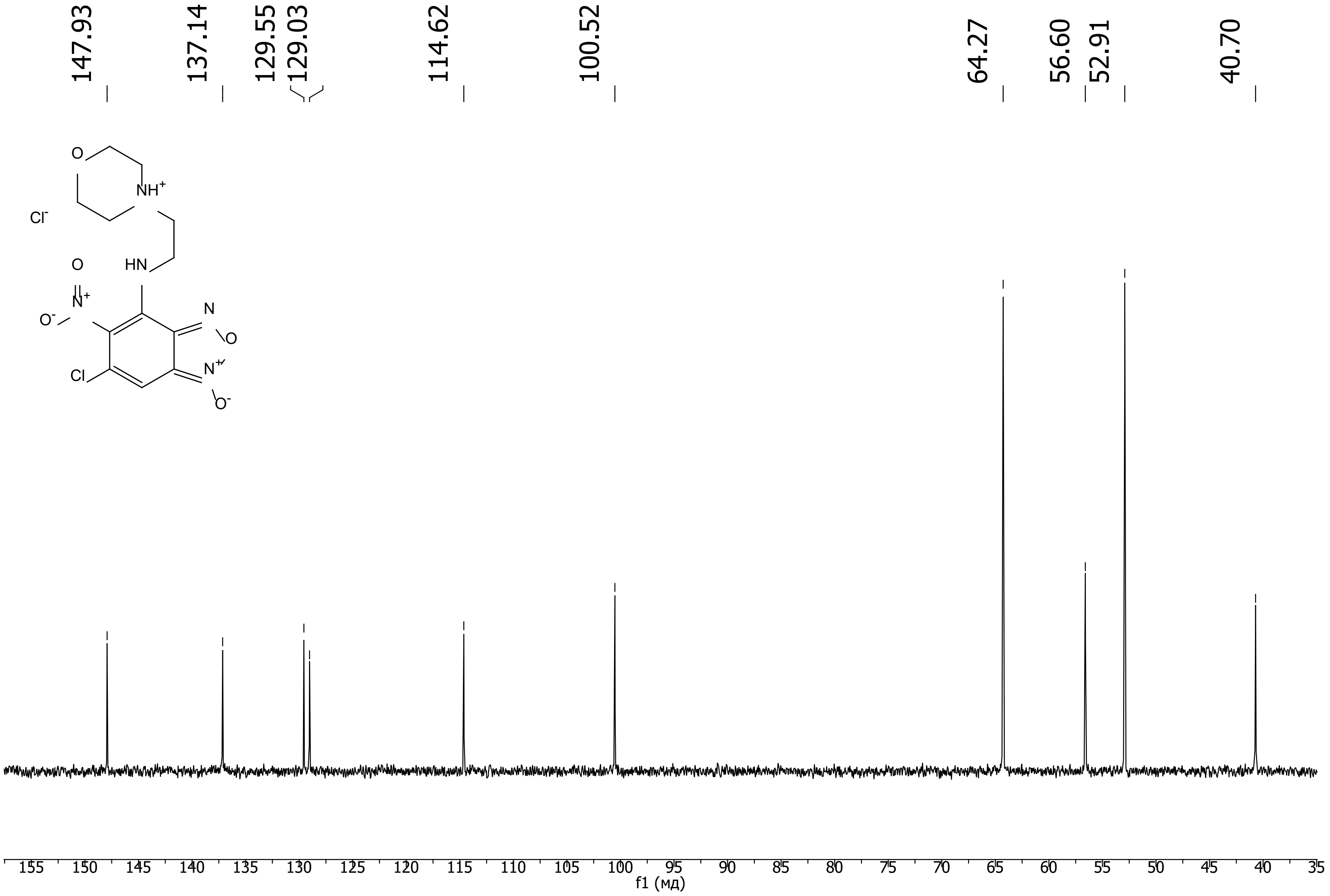

Figure S17. ^{13}C NMR (D_2O, 101 MHz, 303 K) of compound **4d**.

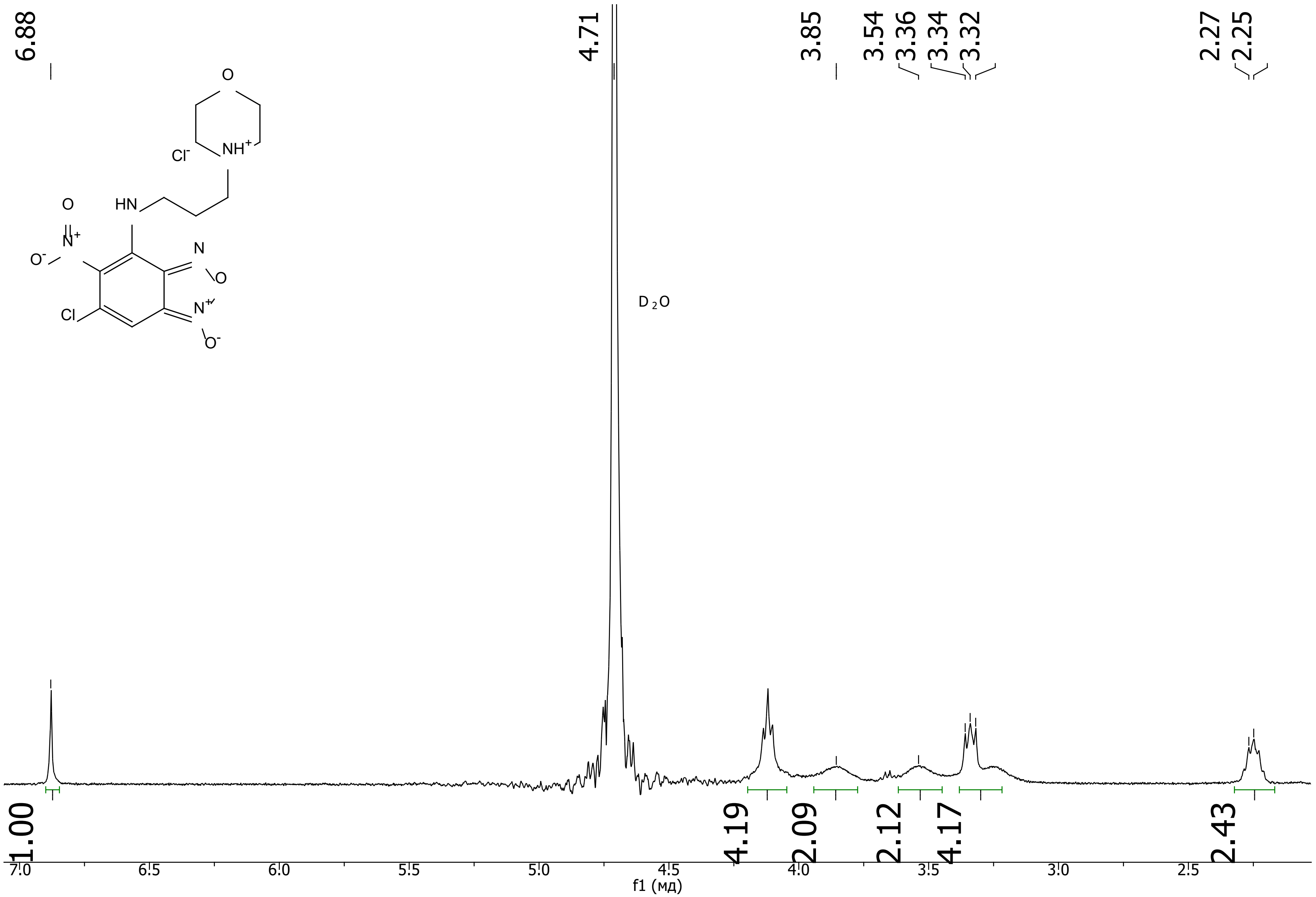

Figure S18. ^{1}H NMR (D_2O, 400 MHz, 303 K) of compound **4e**.

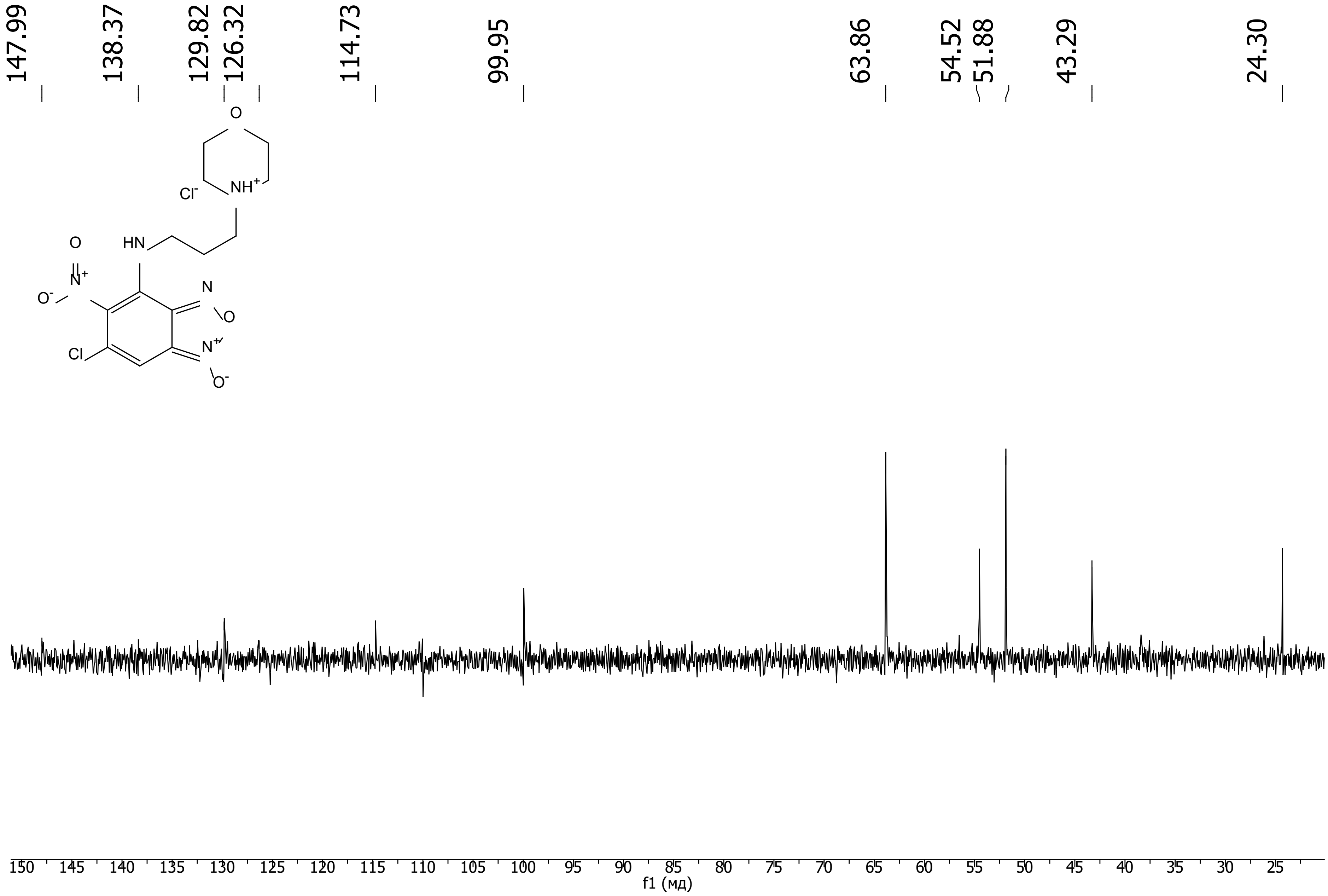

Figure S19. ^{13}C NMR (D_2O, 101 MHz, 303 K) of compound **4e**.

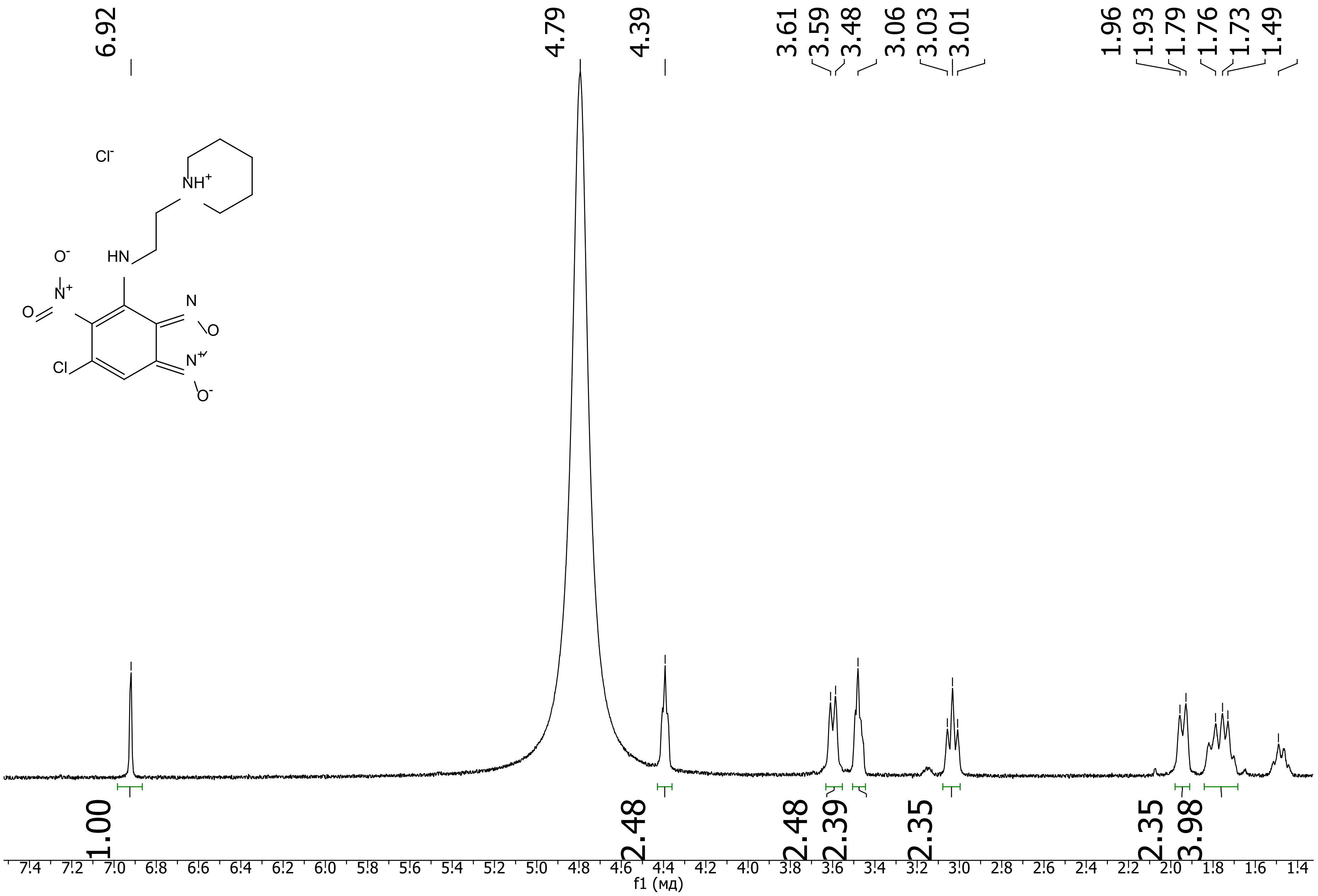

Figure S20. ^{1}H NMR (D_2O, 500 MHz, 303 K) of compound **4f**.

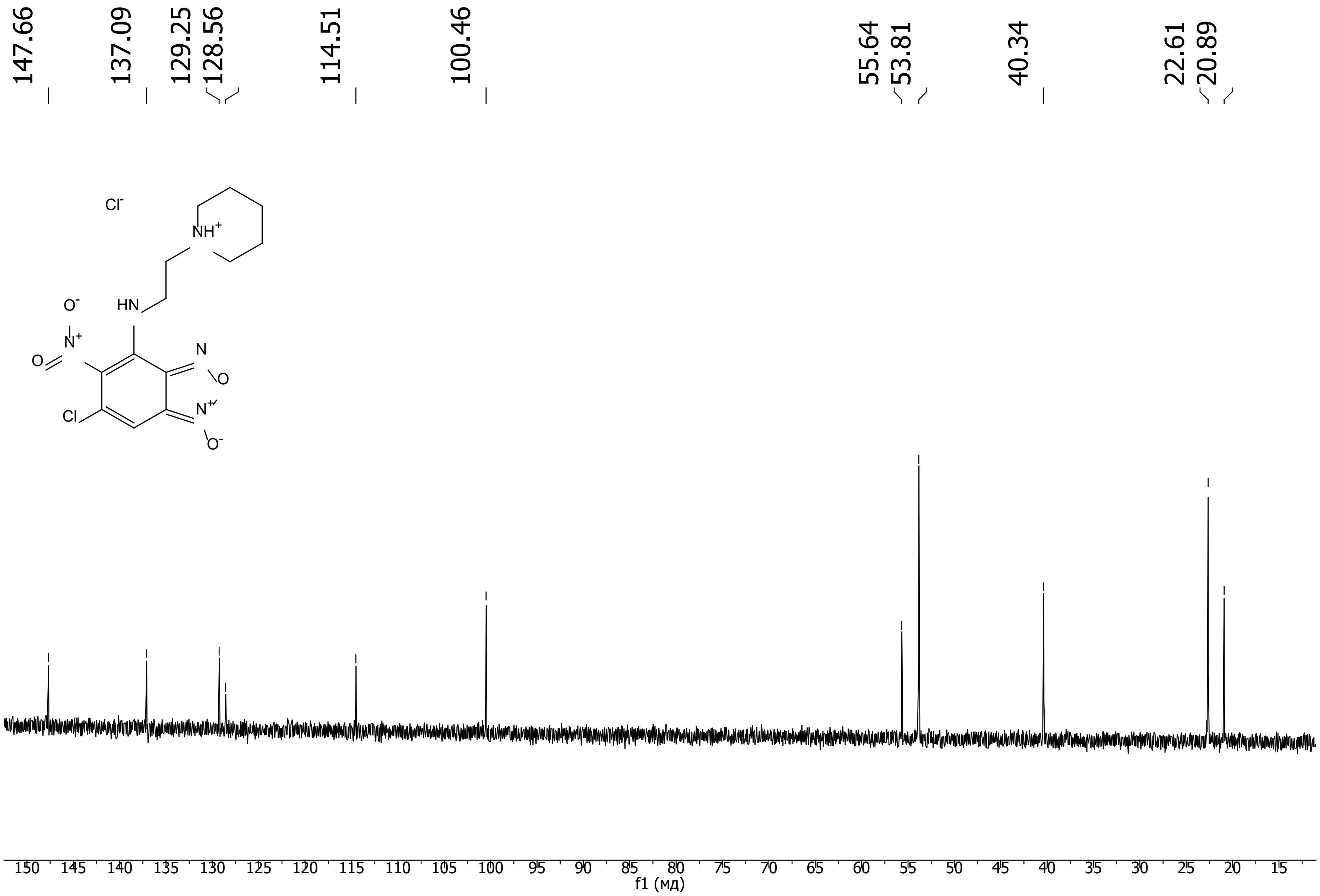

Figure S21. ^{13}C NMR (D_2O, 101 MHz, 303 K) of compound **4f**.

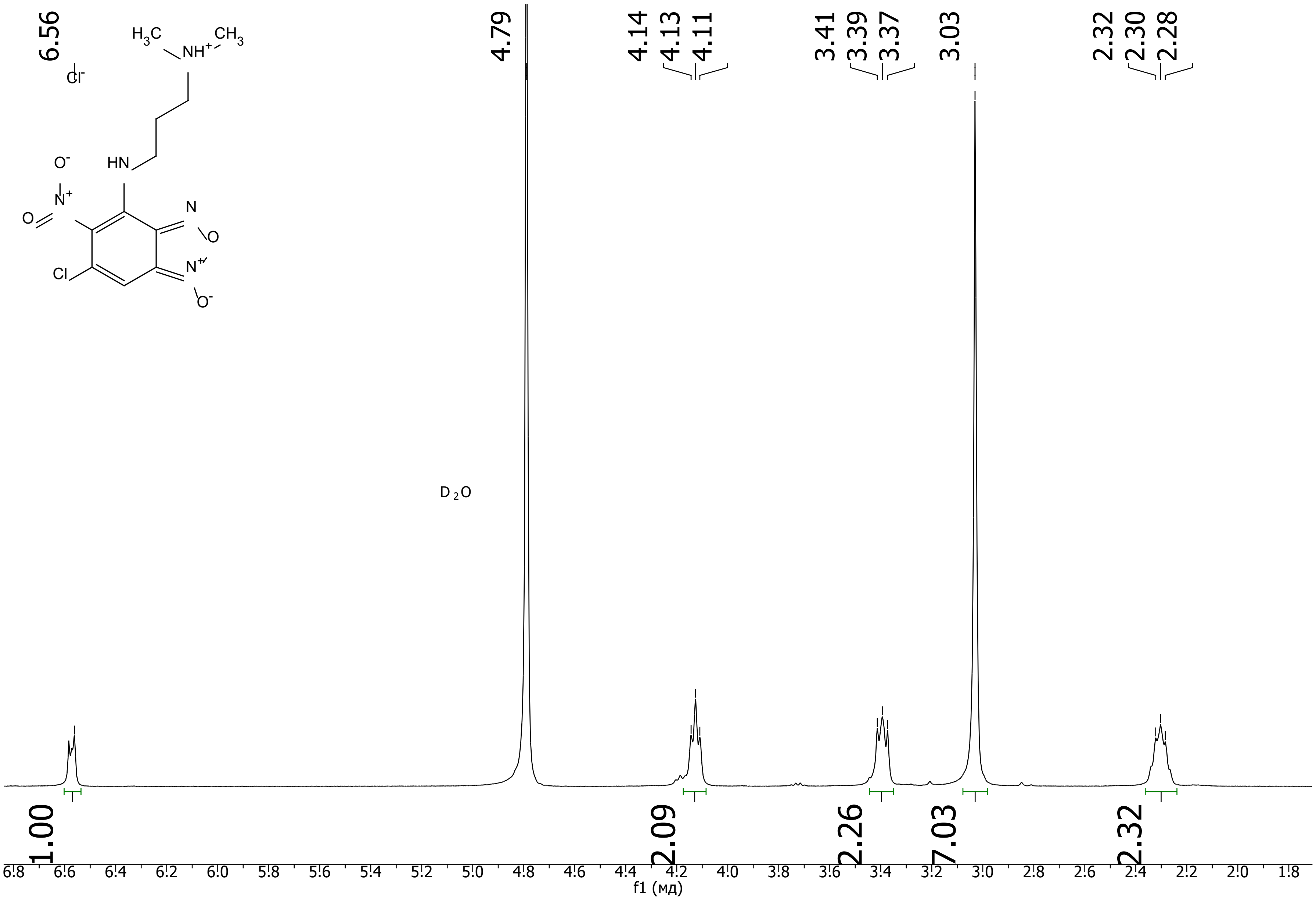

Figure S22. ^{1}H NMR (D_2O, 400 MHz, 303 K) of compound **4g**.

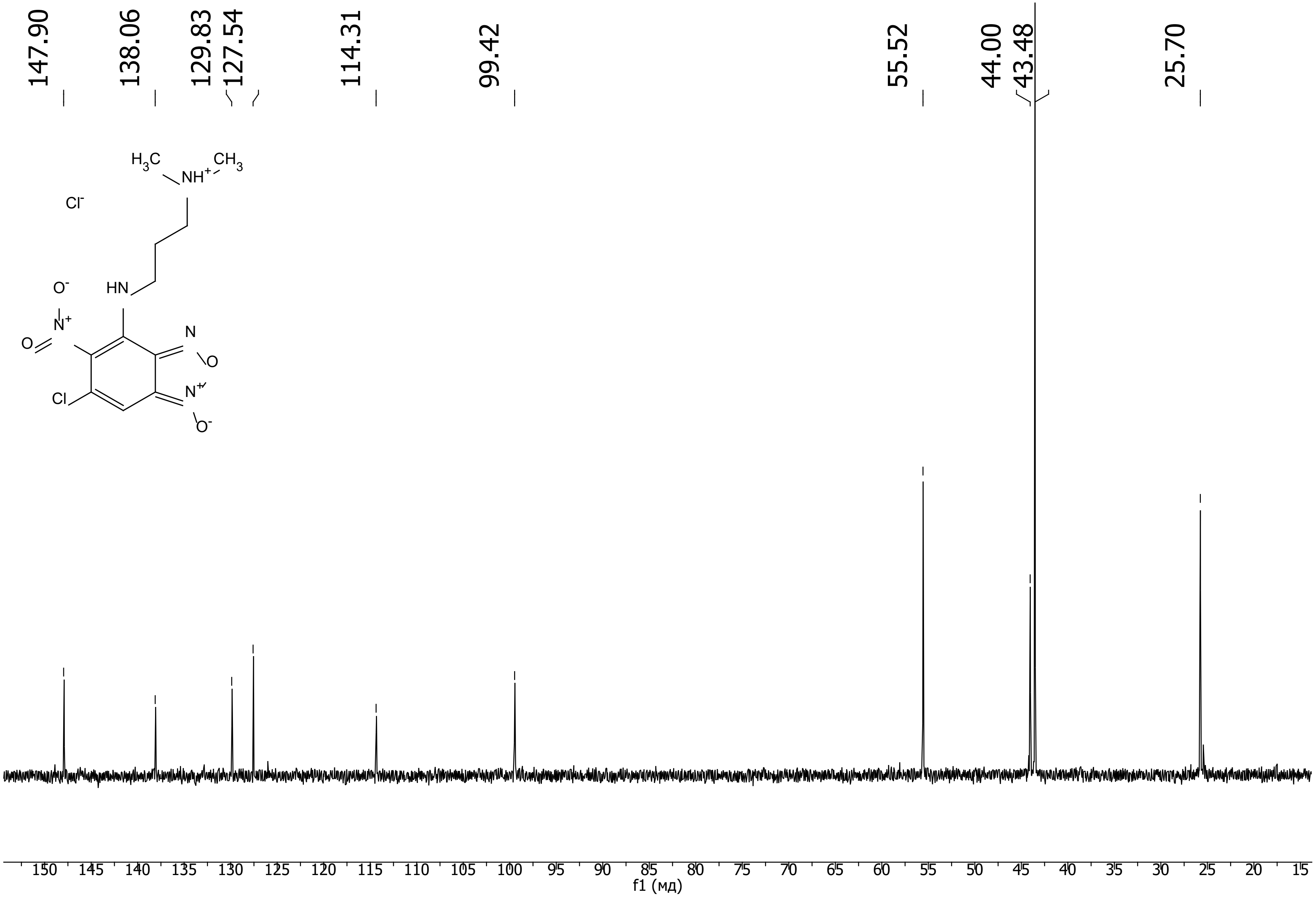

Figure S23. ^{13}C NMR (D_2O, 101 MHz, 303 K) of compound **4g**.

Table S1. Crystal Data and Refinement Details for compounds **3a** and **3d**

	**3a**	**3d**
empirical formula	$C_{15}H_{14}ClN_5O_4$	$C_{12}H_{14}ClN_5O_5$
fw	363.76	343.73
temp.	100	100
cryst syst	Orthorhombic	Monoclinic
space group	$P2_12_12_1$	$P2_1/c$
a (Å)	4.8494(3)	12.5216(5)
b (Å)	10.7547(8)	11.0374(4)
c (Å)	30.200(2)	11.0986(5)
α(deg)	90	90
β(deg)	90	109.840(1)
γ (deg)	90	90
vol (Å^3)	1575.05(18)	1442.85(10)
Z	4	4
density (calcd) (Mg/m^3)	1.534	1.582
Abs.coeff (mm^{-1})	0.276	0.301
F(000)	752	712
cryst size (mm^3)	0.08 x 0.09 x 0.16	0.09 x 0.09 x 0.15
θ range (deg)	1.3, 26.8	1.7, 32.4
index ranges	-5: 6 ; -13: 11 ; -38: 38	-18: 18 ; -16: 16 ; -16: 16
reflns collected	12997	40054
independent (Rint)	3355, 2964 (0.072)	5163, 4709 (0.047)
data/restraints/parameters	3355 /0/ 228	5163 /0/ 208
final R indices		
R_1	0.0581	0.0393
wR_2	0.1537	0.1057
R indices (all data)		
R_1	0.0645	0.0419
wR_2	0.1613	0.1071
goodness-of-fit on F^2	1.096	1.176
largest difference peak and hole (e Å^{-3})	0.86, -0.37	0.61, -0.50

Table S2. Crystal Data and Refinement Details for compounds **3f** and **5**.

	**3f**	**5**
empirical formula	$C_{13}H_{16}ClN_5O_4$	$C_{12}H_{14}ClN_5O_5$
fw	341.76	343.73
temp.	108	100
cryst syst	Triclinic	Monoclinic
space group	P-1	$P2_1/n$
a (Å)	6.9889(2)	6.8055(9)
b (Å)	11.8675(4)	22.365(3)
c (Å)	19.0038(6)	9.1409(11)
α(deg)	77.446(2)	90
β(deg)	85.249(2)	95.645(5)
γ (deg)	85.728(2)	90
vol ($Å^3$)	1530.63(8)	1384.5(3)
Z	4	4
density (calcd) (Mg/m^3)	1.483	1.649
Abs.coeff (mm^{-1})	0.278	0.314
F(000)	712	712
cryst size (mm^3)	0.07 x 0.07 x 0.15	0.08 x 0.09 x 0.16
θ range (deg)	1.8, 29.0	1.8, 26.0
index ranges	-9: 9 ; -16: 16 ; -25: 25	-8: 8 ; -27: 27 ; -10: 11
reflns collected	108037	12558
independent (Rint)	8149, 6382 (0.057)	2735, 1586 (0.197)
data/restraints/parameters	8149 /0/ 415	2735 /0/ 209
final R indices		
R_1	0.0366	0.0957
wR_2	0.1143	0.2003
R indices (all data)		
R_1	0.0521	0.1554
wR_2	0.1262	0.2543
goodness-of-fit on F^2	0.866	1.036
largest difference peak and hole (e $Å^{-3}$)	0.37, -0.40	0.97, -0.78
